# Quantification of biological network perturbations for mechanistic insight and diagnostics using two-layer causal models

**DOI:** 10.1186/1471-2105-15-238

**Published:** 2014-07-11

**Authors:** Florian Martin, Alain Sewer, Marja Talikka, Yang Xiang, Julia Hoeng, Manuel C Peitsch

**Affiliations:** 1 Philip Morris International, R&D, Biological Systems Research, Quai Jeanrenaud 5, 2000 Neuchatel, Switzerland

**Keywords:** Systems biology, Causal network model, Transcriptomics data

## Abstract

**Background:**

High-throughput measurement technologies such as microarrays provide complex datasets reflecting mechanisms perturbed in an experiment, typically a treatment vs. control design. Analysis of these information rich data can be guided based on a priori knowledge, such as networks or set of related proteins or genes. Among those, cause-and-effect network models are becoming increasingly popular and more than eighty such models, describing processes involved in cell proliferation, cell fate, cell stress, and inflammation have already been published. A meaningful systems toxicology approach to study the response of a cell system, or organism, exposed to bio-active substances requires a quantitative measure of dose-response at network level, to go beyond the differential expression of single genes.

**Results:**

We developed a method that quantifies network response in an interpretable manner. It fully exploits the (signed graph) structure of cause-and-effect networks models to integrate and mine transcriptomics measurements. The presented approach also enables the extraction of network-based signatures for predicting a phenotype of interest. The obtained signatures are coherent with the underlying network perturbation and can lead to more robust predictions across independent studies. The value of the various components of our mathematically coherent approach is substantiated using several in vivo and in vitro transcriptomics datasets. As a proof-of-principle, our methodology was applied to unravel mechanisms related to the efficacy of a specific anti-inflammatory drug in patients suffering from ulcerative colitis. A plausible mechanistic explanation of the unequal efficacy of the drug is provided. Moreover, by utilizing the underlying mechanisms, an accurate and robust network-based diagnosis was built to predict the response to the treatment.

**Conclusion:**

The presented framework efficiently integrates transcriptomics data and “cause and effect” network models to enable a mathematically coherent framework from quantitative impact assessment and data interpretation to patient stratification for diagnosis purposes.

## Background

High-throughput measurement technologies provide comprehensive data sets to obtain insight on disease mechanisms and the biological impact of exposure to active substances, such as drugs and environmental toxicants. However, the scientific community faces an ongoing challenge to analyze and interpret these data sets and derive useful insights about the studied biological systems. The analysis of high-throughput expression data typically leads to a list of differentially expressed genes. However, this approach often fails to provide mechanistic insights into the underlying biology. During recent years, researchers addressed the complexity of such data by evaluating them in a relevant biological context [1], whereby genes are grouped based on a priori knowledge such as MSigDB [2]. Sets of genes are then used by algorithms determining their specificity (or enrichment) in a particular experiment [3]. Kathri *et al.*[1] recently reviewed and categorized them into three main successive generations: over-representation analysis (ORA), functional class scoring (FCS) and pathway topology (PT). Unlike ORA approaches that only consider differentially expressed genes, FCS approaches, such as GSEA [2], take into account the entire dataset without applying thresholds. The development of PT approaches was motivated by the increasing evidence that interactions between genes or proteins better describe underlying molecular mechanisms [4]. These approaches allowed for a better use of pathway and network collections, such as KEGG, BioCarta, MIPS [5], the Database of Interacting Proteins (DIP) [6], and the Molecular Interaction database (MINT) [7]. The mapping of genes onto pathway or network representations has resulted in algorithms with better specificity because they account for the topology of the pathway or network [1,8]. Nevertheless, most currently available pathway tools rely on the “forward assumption”, where protein activity changes are assumed to be directly correlated with expression changes of their coding genes [9,10]. This assumption does not always hold [11-13]; furthermore calculations that are based on few genes may lack robustness. Table 1 shows some representative of methodologies relying on this assumption, and their key features.

**Table 1:** **Classification of the different methodologies and some of their representatives (see **[1]**, **[14]** for more exhaustive lists)**

**Reasoning**	**Category**	**Method**	**Qualitative**	**Quantitative**	**Use topology**	**Interpretability**	**Diagnostic Sigs**	**Threshold-free**
	ORA	Hypergometric test [15]	*√*					
		GSEA [2]	*√*	(*√*)		*√*		*√*
	FCS	GSA [52]	*√*	(*√*)				*√*
		PLAGE [16]	*√*	(*√*)				*√*
Forward		CORG [33]				*√*	*√*	*√*
		SPIA [46]	*√*		*√*			
		NetGen [17]	*√*		*√*			*√*
	PT	NetWalker [18]	*√*		*√*	*√*		*√*
		SVM-based [35]			*√*		*√*	*√*
	ORA	RCR [21]	*√*					
		Modified GSA [22]	*√*	*√*				*√*
Backward	FCS	MARINa [20]	*√*					*√*
		NPA [23]	*√*	*√*				*√*
	PT	TopoNPA	*√*	*√*	*√*	*√*	*√*	*√*

For each represented method, their properties are described. The method described in this study, TopoNPA, is to our knowledge the first backward PT methodology. ORA: Over-Representation Analysis, FCS: Functional Class Scoring, PT: Pathway Topology. (*√*): with small modification.

**Figure 1:**
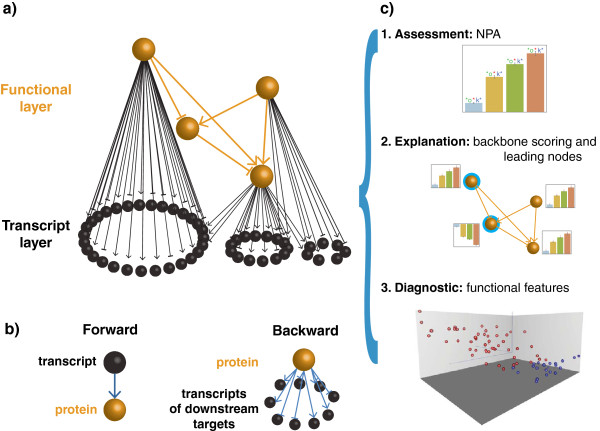
**Our methodology for quantifying the perturbations of biological networks provides a coherent framework for multifaceted results. a)** Biological network model: the functional layer, or the backbone, is shown in orange, whereas the transcriptional layer is shown in black. Each causal edge is directed and signed. A given gene can be modulated by several nodes of the backbone model as depicted by several black arrows linking several nodes of the functional layer to a single node of the transcriptional layer. **b)** The fundamental paradigm shift between forward and backward reasoning; while the former considers the gene transcript abundance as a direct surrogate for its associated protein (or protein function), the latter considers the changes in gene transcription as the consequence of the activity of the upstream biological entity described by a node in the functional layer. **c)** Our new methodology provides a coherent framework connecting Network Perturbation Amplitude quantification (top panel), with mechanistic explanations and identification of the leading nodes in a network (middle panel) and finally the extraction of functional features (i.e., biomarkers) which can be used to stratify patient populations (bottom panel, points are individual samples in the new feature space and colors indicate a different phenotype).

In contrast, the “backward assumption” groups together the genes which have been described in the literature to be regulated by a given molecular entity, referred as to the upstream biological entity (UBE) (Figure 1b). UBEs are as diverse as transcription factors (as in [19] or [20]), protein activities, complexes or bioactive chemical compounds. Relationships between a given UBE and its regulated genes additionally include the sign (inhibition or activation) of the regulation. Such signed gene sets are also called HYPs, standing for “Hypotheses”, as described in [21]. Signed gene sets are leveraged by methodologies such as Reverse Causal Reasoning [21], modified GSA [22], by the network perturbation approach [23] (Table 1). UBEs can be further assembled into networks (Figure 1a), whereby an edge between two entities represents a cause-and-effect relationship, typically an activation or an inhibition. Network nodes may also include entities that are not known to regulate any genes. Thus, these network models have a two-layer structure, as shown in Figure 1a, where the functional level (the UBEs, called the backbone, in orange) is explicitly distinguished from the transcriptional level (the genes, in black). Recently, an ensemble of more than eighty such network models that consist of cause-and-effect relationships between molecular entities and activities (e.g. kinase activation or increased protein abundance) have been published [24-27] and made available to the community for peer-review [28]. The description of the biological context has been manually built into the network models using prior knowledge extracted from both relevant literature and published datasets after a large-scale knowledge mining effort [21]. These networks describe biological processes such as cell proliferation, cell apoptosis and senescence, cell stress, and inflammation. Further biological processes can be described using cause-and-effect relationships from the OpenBEL framework [29]. OpenBEL is an open platform technology designed to collect cause-and-effect statements that can be further assembled into two-layer networks.

To assess qualitatively the enrichment of such networks, one could leverage the results of any (signed) gene set method testing the significance of the UBE’s in the network and subsequently test the over-representation of the significant ones by a hypergeometric or binomial test. Under the backward paradigm, such a method would correspond to an ORA approach (Table 1) with the limitations discussed in [1], such as applying thresholds and ignoring inter-relationships between the entities in the network. In order to score a two-layer network, the entire network can be collapsed into a single signed gene set and an FCS approach that can handle signs (Table 1) can be applied. Martin *et al.*[23] followed this approach, using uniquely defined signs between entities in the network and a fixed reference node. This approach accounts only for a spanning tree of the network and disregards the rest of the topology, hence does not classify as a PT approach as such (Table 1). In a “proof-of-principle” study, using this method, we have quantified the perturbation of a given UBE based on high-throughput data [23] and showed that it correlates with independent assay endpoint. However, the applicability of the method is restricted to causally consistent networks (e.g., no negative feedback loops are allowed), and does not allow for the identification of the key drivers of the network perturbation. Identifying the key mechanisms responsible for the activation or perturbation of a network/gene set is a valuable feature of any methodology, leveraging either the forward or the backward assumption. For example, GSEA approaches extract the leading-genes of a scored gene set to serve as the basis for further interpretation. However many methods (including [23]) do not provide such a layer for interpretation (Table 1).

Gene expression data have being used to derive diagnostic signatures that inform clinicians on disease states or treatment outcomes. Majority of research has involved identifying and scoring signatures that are correlated with a disease phenotype [30,31]. Due to the high number of genes that are measured with high signal to noise ratio and genotypic variability across individuals, gene-level signatures often lack consistency between independent studies. Signatures may also lack biological meaning and interpretability because they are often derived from machine learning approaches that do not include a priori knowledge. A number of studies have shown that network markers tend to be more robust and more accurate when pathways or protein-protein interaction networks were used as substrates to derive predictive signatures [32-38].

The objective of this research was to establish a computational methodology that can integrate gene expression data with a two-layer cause-and-effect network by using its full topology to identify, interpret and quantify the perturbation of the network in response to any treatment. The quantification of the network perturbation (and its significance) extends the concept of differential expression of single genes to the pathways or networks [8] and is of value in fields of toxicology and pharmacology [39], where dose and time response are studied. This approach goes beyond the enrichment approaches used in many pathway/network tools, which are focused on testing a non-enrichment null hypothesis [1,22,40]. The quantification of the network perturbation in response to a treatment enables not only a comparison across several networks, but also a comparison between several treatments on the same network [41]. First, we applied our method to datasets and networks to compare the results qualitatively and quantitatively with the expected outcomes, as well as the results obtained when using other computational approaches. Second, based on one additional dataset derived from a controlled experiment involving the cell cycle, we showed that the key drivers identified by our method are aligned with the expected biology. Third, two additional public datasets were used to quantify the xenobiotic metabolism response to smoke exposure, identify the key drivers and derive a robust smoking exposure signature. The performance of our network-based signature was compared to several recognized computational approaches. Finally, we applied our methodology to study the mechanisms underlying the unequal efficacy of an anti-inflammatory drug in patients suffering from ulcerative colitis and provide a plausible mechanistic hypothesis. Utilizing the underlying mechanisms, an accurate and robust network signature for predicting individual patient responses was generated. The signature over-performed those generated by other computational approaches.

## Methods

### Data

The data used in this study were either obtained from internal experiments that are described hereafter (Additional file 1) or downloaded from the public repositories such as Gene Expression Omnibus (http://www.ncbi.nlm.nih.gov/geo/) or ArrayExpress (http://www.ebi.ac.uk/arrayexpress/) (see Table 2). Raw RNA expression data were analyzed using the affy and gcrma packages of the Bioconductor suite of microarray analysis tools available in the R statistical environment (version 2.14.0). Robust Microarray Analysis (RMA) background correction and quantile normalization were used to generate probe set expression values. The fold-changes and their moderated t-statistics were computed using limma [42].

**Table 2:** Overview of the datasets used

**Data ID**	**Tissue**	**Treatment**
GSE7895	Bronchial Brushing	Smokers (30), non-smokers (20), former smokers (51)
GSE19667	Bronchial Brushing	Smokers (65), non-smokers (45)
GSE12251 & GSE1480	Colonic biopsies	Responders (20) and non-responders (27) pre-treatment
GSE16879	Colonic biopsies	Responders (8) and non-responders (16) pre-/post-treatment
E-MTAB-1842, GSE50254	Rat parenchyma	Main stream smoke (8, 15, or 23 *μ*g nicotine/l) or fresh air. 5 animals per group.
E-MTAB-1272	NHBE cells	2, 4, 6 and 8 hours after washing of CDK4/6 inhibitor. 3 samples per group.
E-MTAB-1311	NRBE cells	Vehicle control or TNF *α* (0.1, 1, 10, 100 ng/ml) × (30 min, 2 h, 24 h). 3 samples per group

GSE identifiers refer to datasets in Gene Expression Omnibus (GEO, http://www.ncbi.nlm.nih.gov/geo/) and E-MTAB identifiers to dataset deposited in ArrayExpress (http://www.ebi.ac.uk/arrayexpress/). The number of samples per group is indicated in parenthesis.

### Network models

Networks models are a representation of the relationships between the biological activities taking place in the considered cellular systems. They are based on information extracted manually from the scientific literature and encoded in the BEL syntax. BEL is a computable format for unambiguously capturing biological entities and their inter-relationships and associating them with external vocabularies and ontologies [29]. The nodes of the networks correspond to molecular biological entities (e.g., protein abundances, protein activities, chemical compounds and gene expression) and also include cellular processes (e.g., apoptosis). The network edges connect two nodes and represent the cause-and-effect relationship between the corresponding entities (e.g., the transcriptional activity of NFKB directly increases the gene expression of BCL2). Edges are directed as a consequence of their causal nature. They are additionally signed, indicating whether the changes (increase or decrease) of the connected nodes have same (→) or opposite (⊣) signs. An ensemble of more than eighty such network models are made available at [28].

In the “backward-causal” paradigm, the changes in the activities of molecular biological processes, the UBE’s, can be inferred based on the changes measured for their causally “downstream” entities, in our case the differential expression of the genes causally affected by considered processes. For example, the activity of CYP1A1 is not measured but its change, between a treated and untreated condition, is reflected in the expression of the genes described to be altered by it (Figure 1a). Another example is the change in the activity of a transcription factor which is deduced from the changes in the expression of its direct targets, and not from the changes in the expression of its mRNA. This paradigm is becoming increasingly popular [21,23,43,44] and among others, “backward-causal” features have been introduced recently in Ingenuity Pathway Analysis software [43]. Using RNAi experimental data, Markowetz *et al.* showed that upstream pathway relationships between unobserved molecular entities can be reliably deduced from downstream measurable entities [45].

This is in contrast to the “forward-causal” approach, where the activity changes of a protein is approximated by the differential expression of its corresponding transcript (see Figure 1a). The number of “downstream” of a typical UBE is between a dozen and several hundreds. Additional details can be found in the Additional file 1.

In a nutshell, our networks models are made of signed gene sets (empty or not) related by signed directed edges. By definition of the two-layer structure there are no edges between genes in the transcript layer as we assume here that relationships between genes are driven by the functional layer. An overview of network models used in this study is given in Table 3. One can observe that the size of the functional layer (a few dozen to hundred) is small as compared to the size of the transcript layer (several thousands of genes). The network models used in this study are further discussed in Additional file 1 and are available in the Additional file 2.

**Table 3:** Statistics for the networks used in this study

	**#Nodes**	**#Edges**	**#Nodes with downstream**	**#Genes involved**
TNF-IL1 *α*-TLR-NF *κ*B (Hs)	116	237	50	3874
TNF-IL1 *α*-TLR-NF *κ*B (Rn)	116	237	50	3874
Cell Cycle (Hs)	127	240	57	8059
Xenobiotic metabolism (Rn)	31	49	20	2668
Xenobiotic metabolism (Hs)	34	53	21	2781

The network edges and nodes can be found in the Additional file 2.

### The topological network perturbation amplitude scoring: TopoNPA

**Figure 2:**
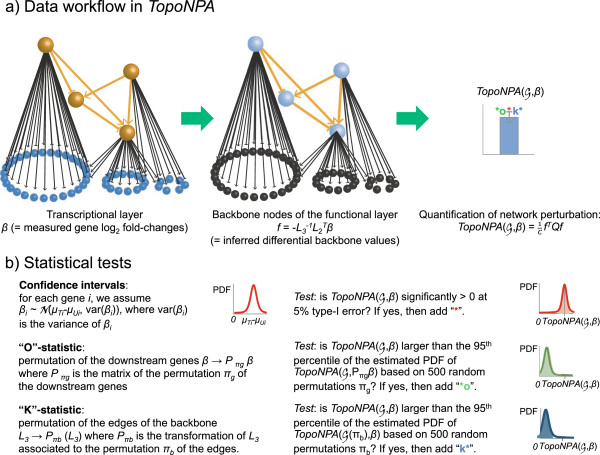
**TopoNPA workflow takes as input gene*log***_***2***_**-fold changes and a fixed network structure. a)** The measured gene *log*_2_-fold changes are used to infer the differential backbone values for which no direct measurements are available. This step relies on the backward assumption. The differential values obtained on the backbone are summarized in a single number quantifying how strong they are and how well those values agree with the signed graph structure of the backbone. **b)** Once the perturbation is quantified, three statistics are computed to assess the significance of TopoNPA with respect to the experimental error and its specificity to the given two-layer network structure.

Our method aims at reducing the high dimensional transcriptomics data by combining the gene expression (*log*_2_)fold-changes into fewer differential backbone values (between a few dozen and two hundred). By definition of the two-layer structure, no measurements corresponding to nodes in the functional layer are available. The differential backbone values will be therefore determined by a fitting procedure that infers values that best satisfy the directional and signed relationships contained in the backbone model (Figure 1a, orange nodes and edges), while being constrained by the experimental data (the gene *log*_2_-fold-changes, *β*) (Figure 1a, black nodes). An overview of the steps involved in the methodology is summarized in Figure 2.

#### Objective and problem statement

Let G=(V,E,σ) be the signed directed graph underlying the biological network (where *V* is the set of vertices, *E* the set of edges, and *σ*:*E*→{+1,−1} is the sign function) and let us assume that  is strongly connected. The set of nodes of the transcript layer is denoted by *V*_0_ and its complement, the backbone, by *V*∖*V*_0_.

For each negatively signed edge, *x*⊣*y* (e.g., between the kinase activities of *AKT*2 and *MAPK*14, *kaof*(*AKT*2)⊣*kaof*(*MAPK*14)), we assume that an additive change of α∈ℝ in the value of *x* results in a change of −*w*(*A*,*B*)·*α* in the value of *y* where *w*(*x*,*y*)>0 is a weight associated with the edge. Similarly for positive relationships, a change of α∈ℝ in the value *x* results in a change of *w*(*x*,*y*)·*α* in the value of *y*. In the absence of further information, the weights are assumed to be 1 as in many network-based approaches (e.g., [35,46]).

If the network model was perfectly representing the data, one could expect the values on *V*, denoted by *f*∈*l*^2^(*V*)^a^, to satisfy all the “cause and effect” statements of the network model and, on *V*_0_, to equal the computed fold-changes. Hence, being given the vector of (*log*_2_-) fold-changes *β*∈*l*^2^(*V*_0_) for the genes in the transcript layer, the optimal fit of the network to the data is computed as the “smoothest” (in the sense of graph regularization [47]) vector *f*∈*l*^2^(*V*) such that its restriction (denoted by |_._) to the transcript layer is equal to the observed fold-changes $f{|}_{V_{0}}=\beta$. Therefore we solve the following optimization problem:

$$\begin{array}{l} {\text{min}}_{f\in l^{2}(V)}\;\underset{x\to y}{\sum }{\left(\;f(x)-\text{sign}(x\to y)f(y)\right)}^{2}w(x,y)\;\\ \;\;\;\;\text{such that}\;f{|}_{V_{0}}=\beta , \end{array}$$

where *w*(*x*,*y*) is the weight associated with the edge bounded by *x* and *y*. This problem relates directly to a Dirichlet boundary condition problem in spectral graph theory [48].

Due to the fact that some biological entities of the backbone are more studied than others in the literature, UBE’s with a lot of downstream genes (edges to the transcriptional layer) will have a very high degree in the graph, as compared other nodes in the backbone. To overcome this issue, the weights associated with the edges *x*→*y* from a backbone node to its *n*_*x*_ downstream in the transcriptional layer (if existing) will be set to $w(x,y)=\frac{1}{n_{x}}$. Equivalently, all the genes have the same importance in describing the UBE’s and therefore the out degree to the transcript layer of any UBE will be 1. This adjustment will value the backbone structure, which is intended to capture the biology and balances the importance of the backbone and the transcript layer (the latter being by far bigger than the former, see Table 3).

#### Solution of the constrained optimization

For *x*∈*v* let *out*(*x*) (resp *in*(*x*)) be the weighted out (resp. in)-degree of the vertex *x*. Leveraging the quadratic nature of the problem, one can show that it is equivalent to:

$$\begin{array}{l} {\text{min}}_{f\in l^{2}(V)}\;f^{T}\left(\text{diag}(\text{out})+\text{diag}(\text{in})-\left(A+A^{T}\right)\right)f\;\\ \;\;\;\;\text{such that}\;f{|}_{V_{0}}=\beta \end{array}$$

where *A* is the signed weighted adjacency matrix defined by

$$A_{\text{xy}}=\left\{\begin{array}{l} \text{sign}(x\to y)\cdot w(x,y)\;\text{if}\;x\to y\\ \;0\;\text{else} \end{array}\right)$$

Even though the problem involves the directed edges only, the quadratic nature of the objective function leads to symmetric matrices and involves the matrix *L*=*diag*(*out*)+*diag*(*in*)−(*A*+*A*^*T*^), which is the signed Laplacian of the undirected graph; hence the directionality of the edges has no more importance at this stage.

Let *L*_3_ denote the sub-matrix of *L* whose rows and columns consist of the backbone nodes, and *L*_2_ the sub-matrix whose rows correspond to the backbone nodes and columns to the genes in the transcript layer. A straightforward differentiation with respect to *f* of the expression above and using the constraint shows that the solution is given by

$$f{|}_{V\setminus V_{0}}=-L_{3}^{-1}L_{2}^{T}\beta$$

As *L*_3_ is weakly diagonal dominant and strictly diagonal dominant for at least one row (as the transcript layer is assumed to be non-empty) and as the underlying undirected backbone graph is assumed to be strongly connected, *L*_3_ is irreducibly diagonally dominant. Therefore, it will be non-singular, ensuring the existence and uniqueness of the solution.

Laplacian and related matrices, like the diffusion kernel on graphs, have been successfully used to prioritize disease genes or to assess pathway enrichment (see e.g., [46,49,50]) where the added value for accounting for the full network topology is demonstrated. Unlike these methods, where the data are mapped directly on a graph, the problem solved here involves an additional boundary constraint as the smoothing is applied to the backbone network only.

#### Quantifying the perturbation

The objective is to summarize into a single number, called Network Perturbation Amplitude, how well and how strongly the inferred values match with the network, with the aim to capture dose and time response of the network, typically in toxicological testing. If all the inferred differential backbone values are high and all the signed edges in the backbone are accommodated by those values (i.e., the objective function for the solution is low), the highest should be the perturbation of the network. Also, as the optimization problem is constrained, the differential backbone values can be relatively high, but still not accommodating well the edge signs. Based on this intuition, we concluded that the perturbation should be a function of the edges and not the nodes alone. Additionally, we should avoid canceling out (“destructive interference”) cumulative signed edge scores (for example when a network with all positive edges contains two subgraphs linked by a single edge, one with all positive values and the other with negative values, one could have a vanishing score while a single edge may be wrong). This situation, where subnetwork perturbations and network perturbation are inconsistent, could typically happen in [23]. Hence, our choice was to consider an “energy” (e.g., quadratic) analogue quantity for each edge. Therefore, the network perturbation amplitude is calculated using the differential backbone values, $f{|}_{V\setminus V_{0}}$ and is defined as a positive number representing the cumulated “energy” of $f{|}_{V\setminus V_{0}}$; i.e., each edge *x*→*y* contributes to (*f*(*x*)+*sign*(*x*→*y*)*f*(*y*))^2^. This score will be consistent with the objective function minimized.

Formally this is defined by the Sobolev (semi-) norm on the graph (*V*,*E*,−*σ*), normalized by the number of edges (*C*=|{*x*→*y*}s.t.*x*,*y*∉*V*_0_|) allowing for more direct comparison between models.

$$\begin{array}{lll} \;\text{TopoNPA}(G,\beta ) & =\frac{1}{C}\underset{\begin{array}{c} x\to y\\ \text{s.t}x,y\notin V_{0} \end{array}}{\sum }{\left(\;f(x)+\text{sign}(x\to y)f(y)\right)}^{2}\; & \;\\ & \;\cdot w(x,y)(*)\; & \; \end{array}$$

Unless explicitly stated, *w*(*x*,*y*)=1 for all the edges of the backbone. Following the argument above, this expression is a quadratic form $\frac{1}{C}\cdot f{|}_{V\setminus V_{0}}^{T}\text{Qf}{|}_{V\setminus V_{0}}$, where *Q*∈*l*^2^(*V*∖*V*_0_)) is defined by

$$\begin{array}{lll} Q & =\left(\text{diag}\left(\text{out}{|}_{V\setminus V_{0}}\right)+\text{diag}(\text{in}{|}_{V\setminus V_{0}})\right)\; & \;\\ & \;\left(-\left(-A-A^{T}\right)\right){|}_{l^{2}(V\setminus V_{0})}\; & \; \end{array}$$

This first step is described in Figure 2a.

#### Vanishing of the TopoNPA score

Let $G_{b}^{-}=(V_{b},E_{b},\sigma_{b})$ be the signed subgraph induced by *V*∖*V*_0_ (the backbone) and for which the sign function is defined by $\sigma_{b}=-\sigma {|}_{E_{b}}$. The signed Laplacian of $G_{b}^{-}$ is exactly *Q*. It follows directly from the Rayleigh quotients that the TopoNPA score is bounded by

$$\begin{array}{lll} 0 & \leq \frac{||\;f{|}_{V\setminus V_{0}}|{|}^{2}}{C}\lambda_{1}\left(G_{b}^{-}\right)\leq \text{TopoNPA}(G,\beta )\; & \;\\ & \leq \frac{||\;f{|}_{V\setminus V_{0}}|{|}^{2}}{C}\lambda_{n}\left(G_{b}^{-}\right)(**)\; & \; \end{array}$$

where $\lambda_{1}\left(G_{b}\right)$ (respectively $\lambda_{n}\left(G_{b}^{-}\right)$) is the smallest (respectively, largest) eigenvalue of *Q*.

Let us focus on the vanishing of $\lambda_{1}\left(G_{b}^{-}\right)$. By definition, a graph is balanced if and only if all its cycles are positive. This property is called “causally consistent” in [23]. Equivalently, $G_{b}^{-}$ is balanced if there exists a partition *V*=*V*_1_∪*V*_2_ such that every edge between *V*_1_ and *V*_2_ is negative and every edge within *V*_1_ or *V*_2_ is positive [51]. It follows from the Matrix-Tree theorem for signed graph, that $\lambda_{1}\left(G_{b}^{-}\right)=0$ if and only if $G_{b}^{-}$ is balanced [51]. This implies in this case that the kernel of *Q* is exactly the subspace of constant functions *f*≡*c* on *V*_1_ and *f*≡−*c* on *V*_2_, for any real *c*. As a formal consequence, if (and only if) the graph is balanced, a TopoNPA score can be zero while the backbone values are piecewise constant as described above; which rarely occurs due to the boundary constraint. The steps involved in building the TopoNPA score are depicted in Figure 2a.

#### Confidence intervals

In order to derive confidence intervals for the differential backbone values and the TopoNPA score, we show that the covariance between the gene (*log*_2_-)fold-changes *β* (which is not the same as the covariance between the genes) vanishes under a weak assumption. Let (*X*,*Y*) be a pair of random variables describing two genes and let us assume that $((X,Y)|\text{Untreated},(X,Y)|\text{Treated})≐\left(X^{U},Y^{U},X^{T},Y^{T}\right)∼N\left(\left(\mu_{U},\mu_{T}\right),\Sigma_{\text{UT}}\right)$. The covariance between the fold-changes, where we have *m*_1_ (respectively, *m*_2_) i.i.d. samples in the treated (respectively, untreated) group, is given by:

$$\;\begin{array}{l} \text{Cov}\;\left(\frac{1}{m_{1}}\overset{m_{1}}{\underset{i=1}{\sum }}X_{i}^{T}-\frac{1}{m_{2}}\overset{m_{2}}{\underset{i=1}{\sum }}X_{i}^{U},\frac{1}{m_{1}}\overset{m_{1}}{\underset{j=1}{\sum }}Y_{j}^{T}-\frac{1}{m_{2}}\overset{m_{2}}{\underset{j=1}{\sum }}Y_{j}^{U}\right)\\ \;=\frac{1}{m_{1}m_{1}}\overset{m_{1}}{\underset{i,j=1}{\sum }}\text{Cov}\;\left(X_{i}^{T},Y_{j}^{T}\right)-\frac{1}{m_{1}m_{2}}\overset{m_{1}}{\underset{i=1}{\sum }}\overset{m_{2}}{\underset{j=1}{\sum }}\text{Cov}\;\left(X_{i}^{T},Y_{j}^{U}\right)\\ \;-\;\frac{1}{m_{2}m_{1}}\;\overset{m_{2}}{\underset{i=1}{\sum }}\overset{m_{1}}{\underset{j=1}{\sum }}\;\text{Cov}\;\left(X_{i}^{U},Y_{j}^{T}\right)\;+\;\frac{1}{m_{2}m_{2}}\overset{m_{2}}{\underset{i,j=1}{\sum }}\;\text{Cov}\;\left(X_{i}^{U},Y_{j}^{U}\right) \end{array}$$

Thus, assuming that there is no second order effect of the treatment (or equivalently homoscedasticity between the treated and untreated groups, *Cov*(*X*^*U*^,*Y*^*U*^)=*Cov*(*X*^*T*^,*Y*^*T*^)=*Cov*(*X*^*U*^,*Y*^*T*^)=*Cov*(*X*^*T*^,*Y*^*U*^)), the above expression vanishes. Consequently, under this assumption, $\beta ∼N(\mu_{T}-\mu_{U},\text{diag}(\text{var}(\beta_{i})))$; which leads to the variance-covariance matrix of the backbone differential values $\text{var}(-L_{3}^{-1}L_{2}^{T}\beta )=L_{3}^{-1}L_{2}^{T}\text{diag}\left(\text{var}\left(\beta_{i}\right)\right)L_{2}{\left(L_{3}^{-1}\right)}^{T}$. As a consequence, the variance of a TopoNPA score can be computed as $\text{var}(h^{T}\text{Qh})=2\text{tr}(Q\Sigma_{2}Q\Sigma_{2})+4\mu_{2}^{T}Q\Sigma_{2}Q\mu_{2}$ (where $h=f{|}_{V\setminus V_{0}}∼N(\mu_{2},\Sigma_{2})$ and $\Sigma_{2}=\text{var}\left(\;f{|}_{V\setminus V_{0}}\right)$). Asymptotically correct confidence intervals are then derived using the central limit theorem (Figure 2b).

#### The companion statistics

Although a TopoNPA score can be highly significant with respect to the biological variation (lower limit of its confidence interval above zero), it does not imply that the gene fold-changes specifically reflect the network structure itself. In order to assess this aspect, two complementary permutation statistics are introduced: the “O” and the “K”-statistics. The ability to distinguish the specifically perturbed networks from the non-specific ones is key as a total of 89 sub-networks have been built to date and can be used simultaneously. These companion statistics quantify the relevance of the information contained in the network model in determining the score (Figure 2b). 

“O” The first one assesses the adequacy of the downstream genes assignment (transcript layer) to the nodes of the backbone model by reshuffling the gene labels in the transcript layer. It tests the null hypothesis that the position of the genes in the transcript layer has no importance in defining the score. Therefore gene labels are permuted and the TopoNPA score is recomputed. This procedure is repeated *B* times (usually *B*=500) and a permutation p-value is derived.

“K” The second statistic assesses the importance of the cause-and-effect relationships encoded in the backbone of the network in extracting the TopoNPA scores. It tests the null hypothesis that the structure of the backbone has no importance in deriving the TopoNPA score. The edges of the functional layer are randomly permuted (together with their signs), differential backbone values are re-computed and TopoNPA is recomputed using the original matrix *Q*. This procedure is repeated *B* (usually *B*=500) times and a permutation p-value is derived.

The upper bound value on (∗∗) states that the highest possible value for the TopoNPA (for unit norm functions) is achieved by the eigenvectors for the largest eigenvalues of $G_{b}^{-}$, which in spectral theory are called the high energy function on the graph. As the highest eigenvalue $\lambda_{n}\left(G_{b}^{-}\right)$ is invariant under the permutations described above, the two statistics can be interpreted as testing if the smoothest function on $G_{b}$ satisfying the constraints given by the experimental data is also a high *l*^2^-norm function and a high energy function (as compared to other random configurations of the network) on $G_{b}^{-}$. Subsequently, the network is considered to be specifically perturbed if both p-values are low (usually 0.05). The TopoNPA results will always be interpreted in light of the three companion statistics: the two permutation p-values and its confidence interval.

#### The leading nodes

To ease the interpretation of a significant perturbation, the major contributors in the sum (∗) can be identified. By sorting the terms in $\text{TopoNPA}=\frac{1}{C}\underset{x\in V\setminus V_{0}}{\sum }(\text{Qf}{|}_{V\setminus V_{0}})(x)\cdot f{|}_{V\setminus V_{0}}(x)$, one compute the contribution of any node *y* as $100\cdot \frac{\frac{1}{C}(\text{Qf}{|}_{V\setminus V_{0}})(y)\cdot f{|}_{V\setminus V_{0}}(y)}{\text{TopoNPA}}$ and rank those accordingly.The key contributors to the perturbation, referred to as leading nodes (Figure 1c, middle panel) are by definition the nodes that make up 80% of the TopoNPA score. It both accounts for the differential backbone values themselves but also to the centrality of the nodes in the functional layer. While 80% is an empirical choice, we have observed that it is usually a promising start to interpret the perturbation and does not preclude, if for example all the contributions are almost equal, to use further ranked nodes. As demonstrated in the next section, the notion of leading nodes is a very useful way to interpret the perturbation; an appropriate understanding of the perturbed biology can only be achieved if the topology of the network and the differential values of the network nodes are equally taken into account.

#### Deriving network signatures

As differential backbone values are obtained through a linear transformation of the fold-changes, individual gene expression profiles can be transformed into backbone values that can subsequently be used for the purpose of classification. For single samples, gene expression profiles are centered, leading to a differential value between the individual profile and the population average. So to map the individual sample data *X* (genes x samples matrix) to the backbone, we simply compute $B=-L_{3}^{-1}L_{2}^{T}X$. This new data matrix will serve as the basis for the classification tasks. As this linear transform does not depend on the data, cross-validation schemes can be performed on the transformed data *B*. The linearity of this transformation ensures that the differential backbone values obtained from the fold-changes are the same as the difference of the average backbone values of the individual mappings. The latter property is important for the coherence of the TopoNPA framework. This step of the methodology depicted in Figure 1c, bottom panel, where points represent samples, colors code for different phenotypes and axes illustrate the backbone nodes.

#### Comparison to quantification/enrichment methods

Comparing gene set/pathway analysis methods in a real experiment is not straightforward because no quantitative performance metrics (like e.g., ROC, Sensitivity, Specificity) are applicable as the “ground truth” is unknown. However, the methods can be compared based on how well their results fit with the existing biological knowledge. This type of assessment is the current best practice in this area [2]. In each use-case, two aspects will be compared to the expected biology: the presence or absence of an enrichment, based on the significance (qualitative) and the dose(/time)-response patterns (quantitative).

As our method is developed with the aim to exploit the specific two-layer structure of cause-and-effect networks, it was compared to the results of methodologies using the same structure, obtained by straightforward adaptations of existing approaches. Those ones belong to three categories [1]: 

*Forward FCS approaches*: Gene-set enrichments methodologies are applied to the set of genes in the transcript layer. Following [22], several combinations of gene-level statistics and gene set enrichment statistics are used. For the gene-level statistics, moderated-t statistics and fold-changes are considered; the former being the canonical choice in FCS enrichment approaches and the latter being the statistics used in TopoNPA. For the enrichment statistics (denoted *ES*, in Additional file 1: Figure S1 and S3), we used the mean, maxmean [52], and GSEA [2].

*Backward ORA approaches*: A straightforward approach to test the enrichment of a two-layer network model is to test the enrichment of each UBE in the functional layer by RCR [21], which is specifically designed for UBE’s and subsequently test the enrichment of the network by a hypergeometric test. The overall “perturbation” is defined as −*log*_10_(*p*−*value*).

*Backward FCS approaches*: Another approach is to test the enrichment of each UBE in the functional layer (as a signed gene set, by multiplying each gene statistics by the sign of the edge from the UBE to the gene) and subsequently test the enrichment of the significant UBE’s by a hypergeometric test. To quantify the perturbation of the functional layer one simply considered the sum of squared enrichment scores of the UBE’s (denoted *SS(ES)* in Additional file 1: Figure S2 and S4). To test the enrichment of UBE’s, the same combinations of gene-level statistics and enrichment statistics described above were used. The results were also compared to our previous methodology, NPA [23].

#### Comparison to signature methods

The comparison between feature selection (i.e., extracting a subset of genes) and feature construction was the main aspect considered. Hence the performance across different machine learning methods (for a fixed set of features) was not the main focus of this work.

Our results were compared to the classification performances of the following computational methods. The first method, not using any of the network information, was the method of the winning team of the IMPROVER Diagnostic Signature Challenge [53,54], referred to as, *tForwardLd*. The first step of the method is to order the features by absolute values of the associated moderated t-statistics. In a second step, the predictors are used one after the other in a LDA model based on this ordering (i.e., LDA based on the first predictor, the two first, three first and so on). At each step an internal cross-validation is computed and the final set of features is selected accordingly. The selection step of this method is included within the cross-validation instances performed in the comparison study to avoid any selection bias in the performance assessment. This methodology was applied on the full set of genes and on the genes in the transcript layer only.

As highlighted in [53] as a take home message of the SBV IMPROVER Diagnostic Signature Challenge, the choice of the base classifier should not impact the prediction performance as much as the choice (either selection or construction) of the features. This observation has led us to choose Linear Discriminant Analysis (LDA), linear support vector machine (SVM), random forests (RF) [55] and, Nearest Shrunken Centroids (NSC) [56] as the base classifiers. In every instance, NSC algorithm cross-validation results were obtained by selecting the shrinking parameter by an internal cross-validation within each cross-validation step.

A more sophisticated methodology, the COndition Responsive Genes (CORG) approach [34] using the UBE’s was also compared. In this approach, one new feature for each UBE is constructed and a classifier is applied on the resulted set of features. Again, the feature construction step is included within each cross-validation step as it depends on the training data.

Finally, LDA or NSC prediction models derived from the genes underlying each UBE individually was build and the best one (based on the cross-validated G-performance (which is the geometric mean between sensitivity and specificity)) was reported.

The extraction of the differential backbone values for individual samples is a purely unsupervised computation and only involves a fixed linear transform of the data $\left(-L_{3}^{-1}L_{2}^{T}\right)$. Therefore, the cross-validation for classifiers based on the backbones values is equivalent to the cross-validation on the original samples for the combination of the mapping to the backbone values and the classifier. All cross-validations are 10-fold cross-validation, averaged over 5 repetitions.

## Results

### TopoNPA distinguishes specific from irrelevant perturbations and enables dose-response calculations

We first evaluated the ability of TopoNPA to capture quantitatively a network perturbation and the companion statistics to distinguish specific from irrelevant perturbations. For that purpose, networks and datasets were chosen where clear biological expectations were available. Those biological expectations will serve as the basis for comparing TopoNPA to other methods.

#### Description of the data and networks

The xenobiotic metabolism network is part of the cell stress model, representing the response to external stressors for non-diseased tissue with a focus on the pulmonary and cardiovascular systems [24]. Representing an unrelated mechanism, the TNF-IL1 *α*-TLR-NF *κ*B network model, including the toll-like receptors (TLRs), interleukin-1A (IL1A) and tumor necrosis factor- *α* (TNF) arms, covers the major signaling pathways that lead to Nuclear Factor- *κ*B (NF *κ*B) activation in response to inflammation [57].

The first dataset (E-MTAB-1842, GSE50254) was derived from a 28-day cigarette smoke inhalation experiment conducted in rats and was expected to show perturbation in both networks, because cigarette smoke is a known inducer of inflammation and xenobiotic metabolism in the rat respiratory tract [58]. We have also used a second dataset (E-MTAB-1311) from rat large airway progenitor cells (RLAK) treated with increasing doses of TNF for 0.5, 2, and 24 hours.

In the first experiment, a dose response of the TNF-IL1 *α*-TLR-NF *κ*B network activation was expected [59]. Similarly, the activation of the xenobiotic metabolism response network was expected to follow a dose response pattern [58]. In the second experiment, the TNF response was monitored by measuring the nuclear translocation of NF *κ*B after treatment at 0.5 hours (data not shown) and led to expect a significant network perturbation amplitude evolving in a dose and time dependent manner. Finally, no xenobiotic metabolism response was expected in this second experiment. The comparison of the results will be performed based on those biological expectations.

#### Comparison to other quantification/enrichment methods

**Figure 3:**
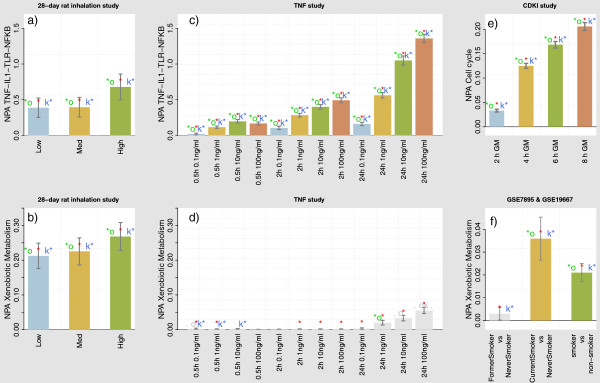
**TopoNPA scores and statistics.** Panels **a)** and **b)**: 28-day rat inhalation study, parenchyma tissue. The two network, xenobiotic metabolism and TNF-IL1a-TLR-NF *κ*B are perturbed across Low, Medium and High dose of cigarette smoke exposure. Panels **c)** and **d)**: TNF treatment on RLAK cells. TNF-IL1a-TLR-NF *κ*B network is perturbed in a dose and time dependent manner, while the xenobiotic network is not perturbed at any dose or time point. Panel **e)** The TopoNPA scores indicate an increased activation of the cell cycle processes as a function of recovery time after inhibitor washout (INH+GM vs. INH+INH). The perturbation is significant for all companion statistics at all time points. Panel **f)** NPA of the xenobiotic network for the comparisons of former smoker vs. never smoker (left bar) and current smoker vs. never smoker (middle bar) in GSE7895. The right bar shows the latter comparison in GSE19667. The significance at 0.05 level of the “O” and “K” statistics are indicated by ‘*o’ and ‘k*’. A grey “.o” or “k.”, indicates a P-value between 0.05 and 0.1. The significance with respect to the experimental variation is indicated by a red star or equivalently can be assessed with the confidence intervals.

The perturbation of the TNF-IL1 *α*-TLR-NF *κ*B network was evident in the rat lung parenchyma when the rats were exposed to smoke as compared to the sham-treated animals (Figure 3a). Similarly, the network was activated in TNF treated RLAK cells in a dose and time dependent manner (Figure 3c). The examination of the p-values revealed a perturbation of the network even with small doses and short TNF treatment times. By contrast, while the biological processes represented in the xenobiotic metabolism network were clearly activated, following an expected dose-response pattern, in the smoke-exposed rat parenchyma (Figure 3b), the companion statistic p-values indicated that they were not specifically perturbed in RLAK cells, by any TNF treatment dosage or time (Figure 3d).

None of the tested method was able to both qualitatively and quantitatively match the biological expectations for those use cases, as summarized in Table 4 (and Additional file 1: Figure S1 and S2). The NPA methodology in [23] was also applied to the TNF-IL1 *α*-TLR-NF *κ*B network and shows a slightly inferior behavior (Additional file 1: Figure S5). Additionally, as the xenobiotic metabolism network is not causally consistent (e.g., due to the negative edge from the node taof(AHR) (“transcriptional activation of nuclear Aryl Hydrocarbon Receptor”) to AHRR (“Aryl Hydrocarbon Receptor Repressor”), this approach was not applicable and further motivated our method that does not assume causal consistency of the backbone.

**Table 4:** Comparison of the qualitative and quantitative behaviors of the network “perturbation” to the expected behavior in the various datasets and studies

**Category**	**Gene-level**	**Enrichment**	**28-day rat inhalation**	**28-day rat inhalation**	**RLAK study**	**RLAK study**	**CDKi study**	**Complete match with**
	**statistics**	**statistics**	**TNF-IL1-NFKB**	**xenobiotic**	**TNF-IL1-NFKB**	**Xenobiotic**	**cell cycle**	**biological expectations**
Forward FCS	t	mean	×/ *√*	×/ *√*	*√*/(*√*)	./ ×	×/ *√*	0
		maxmean	×/ ×	(*√*)/ *√*	×/ ×	./ ×	×/ *√*	0
		GSEA	×/ ×	×/ *√*	×/(*√*)	./(*√*)	×/ *√*	0
	fc	mean	(*√*)/ *√*	(*√*)/ *√*	(*√*)/ *√*	./ ×	×/ *√*	0
		maxmean	***√*/*√***	***√*/*√***	(*√*)/(*√*)	./ ×	×/ *√*	2
		GSEA	×/ *√*	×/ *√*	×/(*√*)	./ ×	×/ *√*	0
Backward ORA	fdr	RCR	×/ ×	*√*/ ×	×/ ×	./ *√*	*√*/ ×	1
Backward FCS	t	mean	×/ ×	×/ *√*	×/ ×	./ *√*	*√*/(*√*)	1
		maxmean	×/ ×	×/(*√*)	×/ ×	./ *√*	*√*/ ×	1
	t	GSEA	×/ ×	×/ *√*	×/ ×	./ *√*	*√*/ ×	1
	fc	mean	(*√*)/(*√*)	*√*/(*√*)	*√*/(*√*)	./ *√*	*√*/(*√*)	1
		maxmean	***√*/*√***	*√*/ ×	***√*/*√***	./ *√*	*√*/(*√*)	3
		GSEA	×/ *√*	×/ *√*	×/ ×	./ *√*	*√*/ ×	1
		NPA	(*√*)/ *√*	na	***√*/*√***	na	na	1
Backward PT	fc	TopoNPA	***√*/*√***	***√*/*√***	***√*/*√***	./ *√*	***√*/*√***	5

A *√* indicates that the result corresponds to the biological expectation, a (*√*) indicate that up to one exception it matches the expectation, a ***×*** indicates that it does not match the expectation, and “.” means that the aspect is unimportant in the case-study. Finally, “na” indicates that the method does not apply. Each use case is assessed for its quantitative (the first symbol) and its qualitative (second symbol) aspects. The comparison scheme is discussed in the material and methods and the table is derived from Figures S1-S5. Overall, TopoNPA behaves as expected and is the only method fully matching the expectations.

In summary, these examples demonstrate that the network perturbation quantifications are able to capture dose and time dependent response, and that, from a qualitative prospective, the developed companion statistics of the method are able to differentiate perturbed from non-perturbed biological processes.

### Leading nodes provide a mechanistic understanding of the perturbation

To assess the ability of our approach to elucidate the driving biological mechanisms in a given network, we applied our methodology to a transcriptomics dataset reflecting the entry to S-phase of the cell cycle, which is a well described mechanism.

#### Description of the data and networks

Normal Human Bronchial Epithelial (NHBE) cells were treated with a CDK inhibitor (PD-0332991), thereby arrested in G1-phase, and then allowed to re-enter the cell cycle by inhibitor washout. In this experiment, the re-entry to the cell cycle was confirmed by Fluorescence Activated Cell Sorting (FACS) analysis of the S-phase cells at 2, 4, 6, and 8 hours after CDK-inhibitor washout (Additional file 1: Figure S9a) and showed a time-response in the increased activity. Transcriptomics data were generated for all time-points with (INH+GM (growth medium only)) and without (INH+INH) inhibitor washout (E-MTAB-1272). We have used our methodology to analyze network perturbation of the cell cycle network model, which is a subnetwork of the previously published cell proliferation model [25] which comprises 127 nodes and 240 edges (see Additional file 2).

#### Comparison to other quantification/enrichment methods

TopoNPA scores for the appropriate fold-changes (INH+GM vs. INH+INH for each time point) are shown in Figure 3e). As expected, the scores reflected a time-dependent increase in the activation of the cell cycle following inhibitor washout, matching the increasing pattern from the FACS analysis (Additional file 1: Figure S9a)). The increase was significant for all companion statistics at every time points, indicating a specific perturbation of the network. In Table 4 (and Additional file 1: Figure S3), we observed that considering the transcript layer as a gene set and using a Forward FCS approach was not efficient. This is due to the fact that more than 8’000 genes are underlying this network (Table 3). Besides TopoNPA, the backward FCS approach based on the “fc/maxmean” enrichment statistics matched the best the expectations. However, it failed to quantify a positive activation of the cell cycle after 2 hours. The cell cycle model contains many negative feedback loops, hence is not causally consistent, preventing the use of [23].

#### Leading nodes interpretation

While the cell cycle subnetwork encompasses all phases of the cell cycle, the leading node analysis (Table 5) were used to identify the key mechanisms that are relevant for re-entry into S-phase. E2F and its binding partner TFDP-1 were the most important nodes in all post-washout time-points that we analyzed, and the activity of the E2F/TFDP1 complex is essential in regulating progression through the cell cycle [60]. In agreement with the literature, several factors known to favor G1 arrest were leading nodes and were predicted to have a decreased activity following washout. These included CDK inhibitors CDKN1A (p21CIP1), CDKN1B (p27KIP1), and CDKN2A (p16INK4A) [61,62] and the retinoblastoma protein (Rb) that forms a complex with E2F, resulting in an inactive or actively repressive complex. Upon phosphorylation by the CDKs, Rb is released from E2F, allowing it to drive entry into S-phase [63-65].

**Table 5:** Rank and direction of change of the leading nodes for the cell cycle network in the CDKi experiment

	**2 h**	**4 h**	**6 h**	**8 h**
	**INH+GM**	**INH+GM**	**INH+GM**	**INH+GM**
taof(E2F2)	2 (+)	1 (+)	1 (+)	1 (+)
taof(TFDP1)	1 (+)	2 (+)	2 (+)	2 (+)
taof(E2F3)	4 (+)	3 (+)	3 (+)	3 (+)
taof(E2F1)	5 (+)	4 (+)	4 (+)	4 (+)
TFDP1	3 (+)	5 (+)	6 (+)	6 (+)
taof(RB1)	6 (-)	6 (-)	5 (-)	5 (-)
G1/S transition of	7 (+)	7 (+)	7 (+)	8 (+)
mitotic cell cycle				
CDC2	8 (+)	8 (+)	9 (+)	9 (+)
THAP1	13 (-)	9 (-)	8 (-)	7 (-)
CDKN1A	12 (-)	10 (-)	10 (-)	10 (-)
E2F2	9 (+)	12 (+)	13 (+)	14 (+)
kaof(CDK2)	15 (+)	11 (+)	11 (+)	12 (+)
kaof(CDC2)	16 (+)	14 (+)	12 (+)	13 (+)
CCNE1	11 (+)	15 (+)	16 (+)	19 (+)
taof(MYC)	18 (+)	16 (+)	15 (+)	15 (+)
CCNA2	10 (+)	13 (+)	17 (+)	25 (+)
taof(FOXM1)		27 (+)	14 (+)	11 (+)
Cell proliferation	19 (+)	19 (+)	18 (+)	18 (+)
kaof(CDK4)	22 (+)	17 (+)	21 (+)	21 (+)
CCNB1		24 (+)	20 (+)	17 (+)
CCNB2		26 (+)	19 (+)	16 (+)
RB1	20 (-)	20 (-)	22 (-)	22 (-)
CDKN1B	21 (-)	18 (-)	25 (-)	24 (-)
CDK4		23 (+)	23 (+)	23 (+)
SKP2			26 (+)	20 (+)
E2F3	14 (+)	21 (+)	27 (+)	31 (+)
CDKN2A	17 (-)	25 (-)	30 (-)	
CCND1		22 (+)	24 (+)	26 (+)
kaof(CDK6)		28 (+)	28 (+)	28 (+)
MYC			29 (+)	27 (+)
FOXM1				29 (+)
ZBTB17				30 (+)
taof(SMARCA4)			31 (-)	
taof(E2F4)			32 (-)	
CDC25C				32 (+)
CDK2				33 (+)
CDC25B				34 (+)

In summary, the TopoNPA could identify and quantify the activation of the cell cycle network upon inhibitor washout, and capture the essential molecular mechanisms involved in the G1 to S transition.

### TopoNPA-based diagnostic signatures provide interpretable and robust predictions

To demonstrate how TopoNPA can be employed to derive robust network signatures, we predicted the smoking status of individuals based on the xenobiotic metabolism network using transcriptomics data from bronchial brushing. As mentioned above, the activation of the xenobiotic metabolizing machinery is the main immediate cellular response to combat environmental stressors. In respiratory tissue, the activation of this cellular defense system is highly sensitive and strictly controlled at the transcriptional level by the regulation of the Aryl Hydrocarbon Receptor (AHR) activity. Therefore, the activation of this cellular defense system may be a suitable marker to capture the actual exposure of the target tissue to environmental stressors such as cigarette smoke. The first data set (GSE7895, [66]) was derived from bronchial brushing of “current smokers”, “former smokers” and “never smokers” samples, as described by Beane *et al*. The second data set (GSE19667, [67]) was generated from small airway epithelium samples obtained by bronchoscopy from smokers and non-smokers.

#### Quantification of the xenobiotic network response

The first step was to compute the TopoNPA scores for the transcriptomics changes between current smoker and never smokers to confirm the activation of the xenobiotic metabolism machinery. For GSE7895, we also compared former smokers with never smokers. The obtained scores were clearly elevated for the current smokers in both data sets (Figure 3f), while the absolute scores of the two data sets were unequal. Such differences may be due to short or long term smoking histories or the distinct cell types which compose the two samples (large vs. small airways) [68]. The former smokers comprised in the GSE7895 data set exhibited a TopoNPA score similar to never smokers probably because the xenobiotic metabolism is no longer activated in former smokers as can be easily seen in the changes in gene expression [66,69].

#### Interpretation based on the leading nodes

The second step in our process was to identify the leading xenobiotic metabolism network nodes congruent with the two data sets.

Despite the difference in absolute TopoNPA score, the leading node lists derived from the two datasets were very similar (Table 6), taof(AHR) (“transcriptional activation of Aryl Hydrocarbon Receptor”) being the most highly ranked leading node. Leading nodes such as Diesel Exhaust Particles, Polycyclic Aromatic Hydrocarbons (PAH) (also found in cigarette smoke), and Particulate Matter represent stimuli that initiate signaling cascades similar to those known to be triggered by cigarette smoke. These include the catalytic activity (“catof”) of the P450 family enzymes (catof(CYP1B1), catof(CYP1A1), catof(CYP1A2)) [70] leading to the production of Reactive Oxygen Species (ROS) [71] as well as taof(AHR) [72], all highlighted as leading nodes. Finally, CYP2E1 activity (oxof(CYP2E1) “oxidase-like activity of CYP2E1”), also known to be activated by cigarette smoking [70,73], is a leading node. By definition of the leading nodes, each of these entities are the processes in the backbone that are both highly perturbed and central in the network and altogether explain the network perturbation.

**Table 6:** NPA leading nodes of the xenobiotic network for the comparison current smoker vs. never smoker, all are positive

**Rank**	**GSE7895**	**GSE19667**
1	**taof(AHR)** (+)	taof(AHR) (+)
2	Reactive Oxygen Species (+)	**Diesel exhaust particles** (+)
3	**Diesel exhaust particles** (+)	**Reactive Oxygen Species** (+)
4	**taof(NFE2L2)** (+)	**NFE2L2** (+)
5	**NFE2L2** (+)	**taof(NFE2L2)** (+)
6	**8-Methyl-IQX** (+)	**8-Methyl-IQX** (+)
7	**AHR** (+)	Polycyclic
		Aromatic Hydrocarbons (+)
8	oxof(CYP2E1) (+)	catof(CYP1B1) (+)
9	Polycyclic	CYP1A1 (+)
	Aromatic Hydrocarbons (+)	
10	catof(CYP1B1) (+)	catof(CYP1A2) (+)
11	catof(CYP1A2) (+)	catof(CYP1A1) (+)
12	CYP1A1 (+)	Particulate Matter (+)
13	catof(CYP1A1) (+)	Soot (+)
14	**Soot** (+)	**AHR** (+)
15		oxof(CYP2E1) (+)

The nodes also extracted by Nearest Shrunken Centroids are shown in bold.

#### Network-based signature extraction and comparison to other methods

We next transformed the individual gene expression data into network differential backbone values. Extracting the xenobiotic-relevant biology with the network backbone enables a supervised mechanism-based separation of the smoker and never-smokers groups. The comparisons were performed as described in the method section and were reported in Table 7. The best performance was obtained by training Nearest Shrunken Centroids (NSC) [56] algorithm on the obtained backbone values, by selecting the shrinking parameter by cross-validation within each cross-validation steps. The resulting models led to very good cross-validation specificity (Sp) and sensitivity (Se) for both datasets. Importantly, classifying one study based on the other led to very robust results (GSE7895 predicts GSE19667: Sp=0.87,Se=0.95 and GSE19667 Predicts GSE7895: Sp=0.90, Se=0.94) (Table 7). The best single UBE classifier was based on the genes underlying 8-Methyl-IQX, which was also a leading nodes (Table 6).

**Table 7:** **Prediction sensitivities and specificities for the two datasets, GSE7895 (** ***D***_**1**_**), GSE19667 (** ***D***_**2**_**)**

**Type**	**Method**	**CV (*D***_**1**_**/*D***_**2**_**)**		***D***_**1**_**→** ***D***_**2**_		***D***_**2**_**→** ***D***_**1**_		**Mean G-perf**
		**Se**	**Sp**	**Se**	**Sp**	**Se**	**Sp**	**Test sets**
All Genes	tForwardLd	0.95/0.86	0.93/0.92	1.00	0.87	0.69	1.00	**0.88**
	NSC	0.92/0.94	0.96/0.93	1.00	0.00	0.98	0.00	0.00
	RF	0.75/0.96	1.00/0.97	1.00	0.31	0.98	0.95	**0.76**
	SVM	0.84/0.94	0.98/0.96	1.00	0.00	1.00	0.00	0.00
Gene in transcript layer	tForwardLd	0.95/0.89	0.92/0.93	0.97	0.91	0.61	0.85	**0.83**
	LDA	0.92/0.94	0.80/0.97	0.62	0.27	0.86	0.40	0.50
	NSC	0.96/0.92	0.93/0.95	1.00	0.00	1.00	0.00	0.00
	RF	0.87/0.96	1.00/0.97	0.98	0.58	0.94	1.00	**0.86**
	SVM	0.88/0.95	0.98/0.95	1.00	0.00	0.78	0.15	0.17
UBE downstream genes	CORG + LDA	0.97/0.94	0.90/0.94	0.83	0.36	0.61	0.55	0.56
	CORG + NSC	0.98/0.95	0.93/0.96	0.97	0.00	0.67	0.30	0.22
	Best LDA (8-Methyl-IQX)	0.96/0.95	0.88/0.96	0.9	0.80	0.80	1.00	**0.89**
	Best NSC (8-Methyl-IQX)	0.96/0.86	0.92/0.98	0.98	0.76	0.86	1.00	**0.90**
Backbone values	tForwardLd	0.97/0.90	0.95/0.97	0.8	0.82	0.84	0.90	**0.86**
	LDA	0.96/0.92	0.94/0.98	0.89	0.80	0.84	0.90	**0.86**
	NSC	0.93/0.93	0.81/0.92	0.95	0.87	0.94	0.90	**0.91**
	RF	0.93/0.91	0.80/0.91	0.97	0.73	0.88	0.85	**0.85**
	SVM	0.93/0.93	0.88/0.91	0.98	0.62	0.88	0.90	**0.83**

The predictions of the samples from the dataset *D*_*j*_ based on the model trained on the dataset *D*_*i*_ are reported in the columns *D*_*i*_→*D*_*j*_. While not systematically having the best cross-validation performance, the predictors based on the backbone values show are more robust behavior when predicting one dataset based on the model trained on the other one. The mean of the G-performances ($=\sqrt{\text{Sp}\cdot \text{Se}}$) over the two independent test sets are shown in the rightmost column and is highlighted if ***>***0.7. The best UBE-based models is chosen based on the mean cross-validation G-performance for the two datasets. The algorithms based on the backbone values leads systematically to good performances. The cross-validation standard errors are reported in Additional file 1: Table S1.

While the cross-validation performances were roughly similar, methods based on the backbone values led systematically to higher accuracies on independent datasets (Table 7).

This showed the ability of network signatures to efficiently handle inter-study bias which is known to greatly affect gene-based signature predictions. To illustrate the effect of the transformation to backbone values, principal component analysis (PCA) was carried out on a) the full set of genes, b) the set of genes underlying the network and c) the backbone values of each individual in the study. As can be seen from Additional file 1: Figure S6, the effect of the smoking phenotype was not the dominant source of variation for the two first cases (panels a and b), and the direction of separation of the smoking group averages were strongly disagreeing. In contrast, the phenotype aligned with the first principal component (66% of the inertia) in the last case (panel c). This indicated that the transformation of the gene expression profiles into differential backbone values matched the smoking phenotype with the biology encoded in the xenobiotic metabolism network and potentially reduced the inter-study bias. Finally, Additional file 1: Figure S6b shows that this property was not inherent in the gene set defined by the transcript layer.

The backbone nodes used by Nearest Shrunken Centroids (last line of Table 7) are all leading nodes (Table 6), with a single exception, “Indirubin”. This shows a striking coherence between the important nodes in the network signature and the mechanistic explanation of the TopoNPA score based on leading nodes.

In summary, TopoNPA ability to establish a quantitative marker for cigarette smoke exposure was verified by applying the method to datasets from different studies. It enabled the derivation of a robust network signature for smoking exposure in the lung, and provided a robust mechanistic explanation, which showed the coherence of the TopoNPA framework. Moreover, the method could extract the meaningful biology from a transcriptomics data set and classify study participants accordingly by reducing the importance of the sources of variations irrelevant to the biology.

### TopoNPA identifies abnormal TLR signaling as a plausible explanation for poor response to anti-TNF ***α*** drug in the treatment ulcerative colitis

Next we applied TopoNPA to derive a plausible mechanistic explanation of the unequal efficacy of an anti-inflammatory drug in patients suffering from ulcerative colitis and to generate an accurate and robust network signature for predicting individual patient responses to the treatment.

#### Inflammatory bowel disease

Inflammatory bowel disease (IBD) is regarded as a disturbance of immune homeostasis in the mammalian intestine [74]. As evidenced by a number of studies, TNF *α* plays a major role in IBD pathology, and TNF-blocking agents have proven an effective therapy to both ulcerative colitis (UC) and Crohn’s disease (CD) [75]. However, the treatment outcome is highly variable; a monoclonal antibody against TNF, Infliximab (IFX), induces remission in only one third of patients with moderate to severe UC [76]. To avoid unnecessary adverse drug effects and needlessly high treatment costs, it is imperative to develop reliable biomarkers to predict treatment outcome [77]. Gene signatures have emerged as powerful tools to predict treatment response [78-84]. These include a five-gene signature that could predict IFX response with high accuracy in one cohort, while lacking cross-cohort robustness [79]. The predictive genes were classified as being involved in the adaptive immune response, yet the biological pathways that mediate the resistance to therapy were not identified. To shed more light on the mechanistic reasons behind these IFX responses, we used the canonical TNF-IL1 *α*-TLR-NF *κ*B network model of NF *κ*B signaling to capture the network response to measured gene expression levels. In addition to TNF signaling, the involvement of both the IL-1 and TLR pathways are well established in IBD pathology [85-88]; in the colonic mucosa, TNF, IL1A, and Toll-like receptor signaling all activate NF *κ*B leading to further expression of inflammatory mediator genes [89]. By applying the TopoNPA approach to publicly available UC transcriptomics datasets, we set out to predict the treatment response in two distinct patient cohorts and investigate the biological pathways involved in the treatment outcome.

#### Quantification of network perturbations

**Figure 4:**
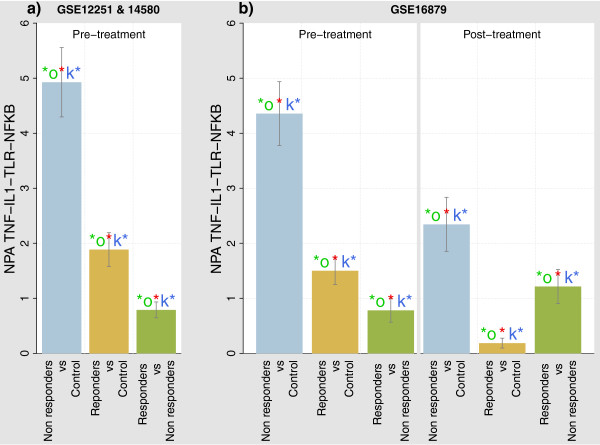
**NPA of the TNF-IL1 *α *-TLR-NF *κ *B network in patients with Ulcerative colitis for the comparisons: IFX non-responders vs. control cases, IFX responders vs. control cases and IFX responders vs. IFX non-responders.** The right-hand side of panel **b)** shows the TopoNPA scores for the samples collected after IFX treatment (GSE16879), while the other bar plots shows the TopoNPA scores before IFX treatment (GSE 12251 & GSE14580 (panel **a)**), GSE16879 (panel **b)** left-hand side). The significance at the 0.05 level of the “O” and “K” statistics are indicated by ‘*o’ and ‘k*’, respectively, while the significance with respect to the experimental variation is indicated by a red star.

We evaluated the transcriptomics profiles of colon samples (GSE 12251 and 14580) from two cohorts of patients prior to receiving their first treatment with IFX for refractory ulcerative colitis [90]. Each patient signature was compared with the average non-responder signature, and the network perturbation of the TNF-IL1 *α*-TLR-NF *κ*B model was used as the input, to find a mechanistic signature differentiating responders from non-responders. The scores for the perturbation of the TNF-IL1 *α*-TLR-NF *κ*B network in IFX responders and non-responders (for the contrasts “non-responder” vs. “control”, “responder” vs. “control” and “responder” vs. “non-responder” in the two cohorts together showed the high perturbation of the network for the non-responder group (Figure 4). We also used another dataset containing gene expression profiles of patient colonic biopsies collected pre- *and* post-treatment (GSE16879). Overall, the non-responder network perturbation was already higher than the responder one. This suggests that the quantitative measure of network perturbation might be indicative of the susceptibility to the treatment.

#### Interpretation based on the leading nodes

**Figure 5:**
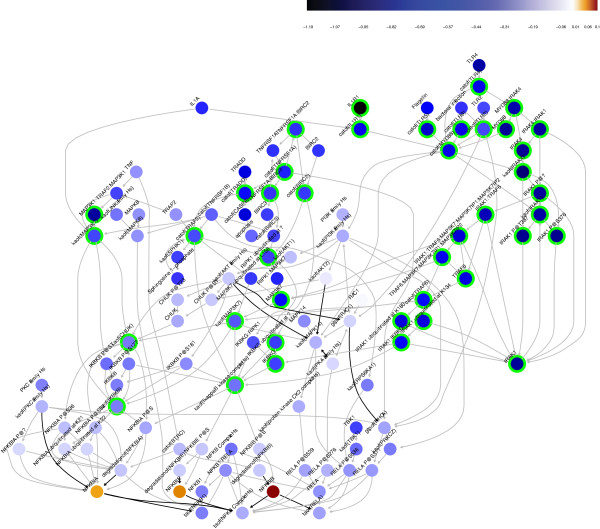
**Differential network backbone values of TNF-IL1 *α *-TLR-NF *κ *B network for the comparison IFX responders vs. IFX non-responders from the two cohorts GSE12251 & 14580, showing stronger network perturbation for the IFX non-responder group.** Blue shading indicates a negative value while red shading a positive one. The leading nodes are circled with green. Grey edges represent an activation while black edges an inhibition. Almost four thousands genes are underlying this network (Table 3).

The initial investigation of the leading nodes that defined the difference between responder and non-responder patients prior to IFX treatment (Figure 5) highlighted the involvement of classical TNFR1- mediated signaling [91]. These nodes were important when comparing all UC patients with healthy controls and responders to non-responders. This is in accordance with previous reports that have demonstrated that TNF *α* gene expression in colorectal mucosa and high serum TNF levels can be used as a predictor for IFX therapy in ulcerative colitis and Crohn’s disease, respectively [92,93]. Interestingly, MYD88-mediated pathways were also predicted to be important when comparing the pretreatment data from responders and non-responders.

Using the dataset containing pre- and post-treatment effects, we observed that, while MYD88 and TLR (namely TLR2-, TLR4- and TLR5-related nodes) remain important nodes to distinguish the non-responders from the responders even after treatment, IL1R1 signaling was predicted to be similar in both subject groups (Additional file 1: Figure S7). Therefore, the underlying biological mechanisms that define the treatment response in UC patients were possibly related to abnormal TLR signaling and did not involve directly IL1R1.

#### Extracting a network-based signature

Standard gene expression-based approaches to predicting the response to IFX from such samples rely on finding gene signatures (i.e., a short list of genes) informing the clinicians of the possible response to the treatment. Arijs *et al.* reported a small gene signature using gene filtering and Nearest Shrunken Centroids that suffers from a lack of robustness when using the gene signature derived from one cohort to predict the response of a second cohort of patients [90] (Table 8). The individual patient backbone values of the TNF-IL1 *α*-TLR-NF *κ*B model were used as the input for deriving a signature differentiating responders from non-responders.

**Table 8:** Prediction sensitivities and specificities for the two cohorts, A and B

**Type**	**Method**	**CV (*A */ *B *)**		***A*** **→** ***B***		***B*** **→** ***A***		**Mean G-perf**
		**Se**	**Sp**	**Se**	**Sp**	**Se**	**Sp**	**Test sets**
	tForwardLd	0.38/0.63	0.80/0.65	0.50	0.82	1.00	0.88	**0.79**
	From Arijs, 2009	Accuracy: 0.92/0.91		0.25	1	Accuracy: 0.71		na
All Genes	RF	0.20/0.82	0.88/0.73	0.25	0.91	0.62	0.75	0.58
	SVM	0.52/0.78	0.85/0.69	0.42	0.82	0.62	0.75	0.63
	NSC	0.48/0.78	0.80/0.58	0.67	1.00	0.69	0.88	**0.80**
	tForwardLd	0.50/0.68	0.83/0.62	0.67	1.00	0.88	0.69	**0.80**
	LDA	0.43/0.90	0.84/0.65	0.42	0.82	0.88	0.62	0.66
Gene in transcript layer	NSC	0.60/0.88	0.79/0.58	0.75	0.73	0.88	0.56	**0.72**
	RF	0.28/0.85	0.88/0.71	0.33	0.82	0.88	0.69	0.65
	SVM	0.52/0.77	0.86/0.69	0.42	0.82	0.88	0.69	0.68
	CORG + LDA	0.33/0.73	0.68/0.62	0.25	0.64	0.75	0.69	0.56
	CORG + NSC	0.62/0.90	0.78/0.60	0.50	0.82	0.88	0.69	**0.71**
UBE downstream genes	Best LDA (MAP3K1)	0.45/0.98	0.78/0.69	0.58	0.91	1.00	0.75	**0.80**
	Best NSC (catof(TLR2))	0.85/0.92	0.80/0.56	0.75	1.00	1.00	0.69	**0.85**
	tForwardLd	0.53/0.82	0.75/0.75	0.50	0.64	1.00	0.62	0.68
	LDA	0.75/0.75	0.76/0.75	0.50	0.73	0.88	0.56	0.65
Backbone values	NSC	0.98/0.98	0.75/0.67	0.92	0.82	1.00	0.62	**0.83**
	SVM	0.78/0.78	0.40/0.77	0.91	0.50	0.69	0.88	**0.73**
	RF	0.75/0.82	0.62/0.78	0.82	0.75	0.75	0.88	**0.80**

The predictions of the samples from the cohort A (B respectively) based on the model trained on the cohorts B (A respectively) are reported in the columns *A*→*B* (*B*→*A* respectively). The mean of the G-performances ($=\sqrt{\text{Sp}\cdot \text{Se}}$) over the two independent test sets are shown in the rightmost column and is highlighted if ***>***0.7. The best UBE-based models is chosen based on the mean cross-validation G-performance for the two datasets. The algorithms based on the backbone values leads to good performances for a majority of algorithms. The cross-validation standard errors are reported in Additional file 1: Table S2.

Comparisons were performed following the scheme described in the method section above were reported in Table 8. NSC combined with the genes underlying a single UBE and NSC based on the backbone values as the features for learning led to the best prediction performances across cohorts (Table 8). As in the previous example, performance results showed a more robust behavior of network-based features as compared to gene selection, for the majority of the learning algorithms used. Again, this is in line with the results of the IMPROVER Diagnostic Gene Signature challenge [53], where it is argued that features selection or construction are more important that the base learning algorithm chosen. The best predictions based on a single UBE were obtained using the downstream genes of catof(TLR2) (“catalytic activity of TLR2”), which is one of the leading nodes (Figure 5) and is involved in the TLR-signaling.

As observed in the previous use-case, the inter-study robustness might be explained by the alignment of the responsiveness status with the first principal component (94.7% of the total variance) of the individual backbone values; as opposed to the behavior of the principal components based on the gene expression data (Additional file 1: Figure S8).

## Discussion

We have presented several biological and clinical applications of TopoNPA, a novel quantitative approach that uses knowledge-based cause-and-effect biological network models describing complex processes involving thousands of genes. As an input TopoNPA requires high-throughput transcriptomics data obtained from appropriately designed and executed studies, and a network model relevant to the observed biological response. Our method provides a coherent mechanism-based framework for assessing and quantifying the resulting perturbations of the network as required in a dose-response toxicity setting. It also guides mechanistic interpretation at node-level resolution in a fully coherent way with the perturbation itself. The relevance of the mechanistic interpretation of the network perturbation has been demonstrated in two controlled *in vitro* studies (CDKi and TNF experiments), and applied to two *in vivo* studies. In addition to the quantification of the perturbation and its interpretation, our framework enables the establishment of coherent, efficient and robust diagnostic classifiers based on individual sample network perturbations. We have shown how to use the established biology of xenobiotic metabolism to stratify a human cohort of smokers and non-smokers. The full potential of this powerful approach for deriving mechanism-based interpretation and diagnosis has been further demonstrated in the context of an *in-vivo* study of ulcerative colitis patients, where a testable hypothesis has been proposed regarding the individual response to the drug Infliximab (see below).

### Network perturbation amplitude methodology

Overall, the TopoNPA approach belongs to the class of backward PT methods (Table 1), and is threshold-free because it does not require any filtering of the gene expression data based on expression value or statistical significance. In our method, the absence of any fold-change between conditions can be as informative as high fold-changes. It provides a single framework for quantitative and qualitative evaluation of the amplitude of perturbation of a network.

It applies to two-layer cause-and-effect models without any assumption beyond the underlying undirected graph to be strongly connected. In contrast to the approach in [23], the quantification of network perturbations as a positive-definite quantity (quadratic in the *β*) allows for causal inconsistency, does not require the choice of a reference node, uses fully the topology of the network and avoids canceling out “destructive interference” in cumulative signed amplitudes (for example when half of the network is positive, and half is negative). Our choice was to consider an “energy” analogue quantity (Figure 1c, top panel) rather that a signed value as used by Martin *et al*. As a particular case, if the functional layer was made of a single node (with a self-loop), then the backbone differential value of that node would equal the NPA score in [23].

TopoNPA enables the quantification of the network response to a treatment, which is a key feature in systems toxicology. Attempts to adapt existing gene set-based methods cannot exploit fully the two-layer network structure as either the quantification or the qualitative assessment of the perturbations computed in the different studies did not match the biological expectations (Table 4).

Our framework offers the possibility to identify key contributors of the perturbation in the network using the leading nodes (Figure 1c, middle panel), which have been shown to be pertinent in the positive control experiment (CDKi experiment). This aspect is not covered in our previous methodology [23] as the network was aggregated in a single UBE structure and subsequently scored. An even deeper investigation is possible by defining a similar notion of leading genes for any (leading) node in the network. However, we believe that the gene expression is the consequence and not the driver for the biological processes of the network, and therefore this was not be systematically looked at. Furthermore, the modern view of network biology implies that the essence of biological processes does not lie in the individual behavior of genes, but rather in their collective action, which is precisely captured by the biological network models [4,41,94].

### Network-based Signatures: constructing vs. extracting features

We have shown in two cases that our approach has led to accurate and, importantly, robust classification of individual samples. Even if our goal was not to develop a purely machine-learning algorithm, it clearly appeared that an appropriate choice of the network model led to remarkable performances in classification tasks that is over-performing other approaches and the results published by the authors of the original studies. TopoNPA enabled to build network features that leads to more robust classification results, almost independently of the learning methodology used. The key aspect for this behavior is that gene expression profiles are used to construct (as opposed to extract) new features in a surjective way by a *data independent* linear mapping (Figure 1c, bottom panel). As the dimension of the backbone is far lower than the dimension of the underlying gene space, it is expected that our network signatures will be robust. Additionally if the phenotype of interest is associated with the biology encoded in the functional layer of the network, the class separation is expected to align with the main variation of the differential backbone values. This behavior is apparent in the PCAs constructed on the backbone values: the inter-class variability is aligning with the main direction of variation. Additionally, the transformation seems to reduce the inter-study bias. Another benefit of this linear transformation is to move away from the so-called “ *p*>>*n* problem”, where there are far more variables than samples in a dataset and hence lead to more robust diagnoses. Gene-sets or network have also been leveraged in classification problems based on gene-expression data [33-35]. However the enrichment of a given gene-set and its ability to provide discriminant score rely usually on independent algorithms. Also, this aspect is not covered in our previous methodology [23].

This application revealed a promising potential for our approach in the context of personalized medicine: personalized diagnosis based on the classifier to decide the administration of the Infliximab treatment. Also, the smoking network signature derived could serve as an individual marker of smoking exposure in a clinical trial.

### Network-based biological explanation and hypothesis generation

The analysis of UC patient data from responder and non-responder subjects prompted the generation of a network-level hypothesis. Based on our analysis, TNFR1A-signaling seems to be an important node in defining the difference between the responders and non-responders before and after anti-TNF treatment. This suggests that the treatment fails to fully restore normal TNF signaling in the colonic mucosa of non-responders. However, a recent study has shown that TNF-expression returned to normal at week 30 in both IFX responders and non-responders [95]. Therefore, the stimulated TNF signaling is not the only explanation for poor treatment outcome in non-responders. Our results indicate that the underlying biological mechanisms that define the treatment response to TNF blocking agents in UC patients might be related to abnormal MYD88-signaling. MYD88 has been found to be involved in IBD pathology [96,97] and the involvement of MYD88 in response to TNF-blocking drugs has been demonstrated in studies on rheumatoid arthritis (RA) patients [98]. Our analyses further showed that MYD88 and TLR (namely TLR2-, TLR4- and TLR5-related nodes) remained important nodes to distinguish the non-responders from the responders even after treatment. A recent study by Toedter *et al.* showed that the genes involved in intestinal epithelial barrier defense were differentially expressed in the responder and non-responder colonic mucosa following Infliximab therapy [95]. Members of the TLR family are involved in the injury of the intestinal epithelial barrier, and their abnormal function could lead to defects in mucosal barrier defense in the non-responder population [99,100]. The identification of a high frequency of peripheral T-regulatory cells as a predictive marker for Infliximab treatment response provides additional support for the involvement of abnormal TLR signaling [101-103]. Triptolide, an active component isolated from the Chinese herb *Tripterygium wilfordii* with anti-inflammatory and immunosuppressive properties, has been proposed as an alternative compound for the treatment of CD. Interestingly, the favorable effect was shown to be mediated by targeted inhibition of TLR2/TLR4 signaling in the IL10-/- CD mouse model, and in cultured colon tissue samples from CD patients [97]. Finally, Gewirtz *et al.*, discovered that patients from a specific ethnic background with deficient TLR5 were protected from developing CD, advocating the pharmacological inhibition of the TLRs as an alternative to anti-TNF compounds to treat IBD [104]. Such an approach has already been applied for the treatment of RA, where TLR2/TLR4 antagonists have proven beneficial in treating an autoimmune disorder [105].

On-going research in our group is currently using several network models in conjunction first, to develop methods of assessing the biological impact of individual substances, or a set of substances on a biological system, as we have discussed in [41] and second to improve the performance of the backbone-based classifiers.

## Conclusion

The methodology proposed in this study provides a coherent framework going beyond the work of [23] (Table 1, last two rows) to handle the two-layered structure of cause-and-effect networks (Figure 1a). Firstly, it quantifies network perturbations using differential gene expression in conjunction with the explicit network topology and tests a non-perturbation null hypothesis (Figure 2); hence it positions itself as a backward-based PT methodology (Table 1). Secondly, our methodology calculates the perturbation of the network by inferring differential values for each node of the functional layer of the network model, referred to as differential backbone values. Those quantities facilitate the biological interpretation by decomposing the amplitude of the perturbation and thereby identifying candidate key nodes of the functional layer. Thirdly, it further enables the calculation of these differential backbone values at sample level, which serves to build a knowledge-driven classification of individual patients. Such classifying signatures, which benefit from the mechanistic interpretation of the differential backbone values, can be used to classify individual patient samples. To the best of our knowledge this is the first approach that provides, in a systematic manner, the ability to fully exploit the information provided in two-layer causal models (i.e. using explicitly the full signed network topology), that serves simultaneously for quantitative perturbation assessment, biological interpretation and diagnostic signature extraction. Here we show that using our biological network models to quantify individual patient responses through a network model relevant to the phenotype of interest is a biologically meaningful, interpretable, robust and effective way of deriving network-based signatures.

As a summary, a novel network-based methodology for quantifying perturbation, interpreting data and for deriving network signature was presented. It fully exploits the specific structure of two-layer cause-and-effect network models, made of a functional layer and a transcript layer. The application of our methodology has provided insight into molecular mechanisms by capturing the perturbed network components with high specificity, and has led to robust signatures for diagnosis. The successful application of our quantitative method has clearly demonstrated the potential of TopoNPA in systems biology and systems toxicology.

## Endnote

^a^$l^{2}(A)=\left\{\;f:A\;\to \;\mathbb{R}|\underset{a\in A}{\sum }f{\left(a\right)}^{2}<\infty \right\}\;$ which, for a finite set *A*, is isomorphic to ${\mathbb{R}}^{N}$, where *N* is the cardinality of *A*.

## Competing interests

The authors declare that they have no competing interests.

## Authors’ contributions

FM developed and implemented the methodology. AS and YX contributed to the methodology development. MT did the biological interpretation of the results. JH and MCP supported the project. All the authors contributed to the writing of the manuscript. All authors read and approved the final manuscript.

## Supplementary Material

Additional file 1 **Supplementary information on network models and data.** All supplementary figures and tables are included in this additional file. Click here for file

Additional file 2 Two-layer causal network models used in this manuscript, in excel format. Click here for file
