# Supplementary material for: Quantification of biological network perturbations for mechanistic insight and diagnostics using two-layer causal models
Source: BMC Bioinformatics. 2014 Jul 11;15:238. doi: 10.1186/1471-2105-15-238 (PMC4227138; doi:10.1186/1471-2105-15-238)
Supplement: Additional file 1 — Supplementary information on network models and data. All supplementary figures and tables are included in this additional file. [file 1471-2105-15-238-S1.pdf]

Supplementary Information - Quantification of biological network perturbations for mechanistic insight and diagnostics using two-layer causal models

Florian Martin*, Alain Sewer, Marja Talikka, Yang Xiang, Julia Hoeng and Manual C. Peitsch

*Correspondence:
florian.martin@pmi.com
Philip Morris International, R&D, Biological Systems Research, Quai Jeanrenaud 5, 2000, Neuchatel, Switzerland
Full list of author information is available at the end of the article

Supplementary Text

Network Models

The backward assumption, which is based on the concept that gene expressions are the consequences of upstream interactions between biological entities represented by network nodes (such as enzymatic activities) requires a significant effort in mining and organizing the knowledge to build “cause and effect” network models. The results presented in this study represent a step forward in network integration and have clearly shown the potential of TopoNPA to structure the gene expression profiles into backbone differential scores in a relevant and robust manner. The networks built to date are pioneering a novel approach to model cellular processes at the molecular level by representing knowledge in a computable format using the Biological Expression Language (BEL) syntax. The Open BEL framework is an open source initiative [1] which aims to promote the BEL syntax as a standard to capture biological knowledge.

The description of molecular processes in terms of network models introduces several innovative features and opens new perspectives for describing biological processes at system levels. First, as a consequence of the BEL semantics, the fundamental entities in the network models are not exclusively the concentrations of the relevant molecules and their variations, but also include their functions and their perturbations. The functional layer is the result of assembling multiple fundamental entities using literature-supported causal relationships, with the goal of covering one specific cellular function, such as cell cycle, xenobiotic metabolism or NFkB signaling. As a result, the integration of experimental data in the context of such a network model naturally provides a “mechanistic” description, as the effects of the experimental treatment contained in the data are described in terms of fundamental and causally connected functional entities underlying the cellular process potentially associated with the observed phenotypes.

Second, the adoption of the backward-causal paradigm in our network models enables the distinction between a “functional layer”, containing the actual biological mechanisms, and a “gene transcript layer” representing the experimentally measurable consequences of the network when using transcriptomic technologies (Figure 1b). This architecture is characteristic of the combination of the network backbone and backward-causal approaches, which constitute two essential features introduced

in this study. Previous works in molecular systems biology used these components partially and separately when integrating prior knowledge into the analysis of transcriptomic data. For instance, the use of protein-protein interaction (PPI) networks in this context is pragmatically based on the "forward" approach (Figure 1a), even though variations in transcript levels do not always translate into a similar variation in protein abundances or activities. Also, PPI networks do not contain the mechanistic aspect discussed above, as their nodes do not specifically represent functional entities whose assembly describes cellular processes such as proliferation or stress. Similarly, most existing backward-causal approaches do not introduce relationships between their upstream entities, in opposition to the complex "functional layer" of the network models used in this study. In most of the cases, a large collection of potential upstream entities are tested independently of one another, and their likelihood is scored individually by an enrichment statistic applied to differential gene expression values [2–4]. A step forward is achieved by the Network Component Analysis (NCA) where several upstream entities are allowed to compete with one another to best model the cause of the observed gene expression values [5]. TopoNPA methodology goes even further by using all the structure contained in the directed and signed networks of upstream entities constituting the "functional layer".

Another important feature of our biological network models is their clearly defined boundaries, which are usually absent in PPI networks. Indeed, a direct physical/chemical interaction between two proteins, as captured in a PPI network, is much less dependent on the tissue context than the functional activity of a protein, which may strongly depend upon the presence of co-factors whose abundance can vary across tissues. This highlights the fact that the relationships in the "functional layer" have a specifically defined context as evidenced by the supporting literature. For example, the functional layers for the Xenobiotic and Cell Cycle models were made specifically for healthy lung tissue, whereas the TNF-IL1a-TLR-NFκB network model was made generically. These considerations clearly indicate that the context-dependent, mechanistic and backward-causal biological network models used in this work constitute a powerful knowledge based substrate for integrating transcriptomic data.

Defining the Biological Content of Network Models

The biological content of the network models is defined during their building phase, which is multi-step iterative process involving human expert knowledge and data-driven machine-assisted techniques. First, guided by a survey of relevant scientific literature into the signaling pathways relevant to the process of interest (e.g., proliferation, stress, or inflammation), a team of experts define the biological boundaries of the network. Cause-and-effect relationships describing these pathways are extracted from the literature and the Selventa Knowledgebase which nucleates the network with assertions derived from the relevant cell types and/or experimental contexts. Second, gene expression data obtained from experiments where the process of interest has been stimulated are analyzed using Reverse Causal Reasoning (RCR, [4, 6]). RCR is a "backward-causal" approach that explicitly integrates Selventa Knowledgebase, taking gene expression profiling data as an input and producing qualitative statements for the activity states of biological entities according

to statistical and biological criteria as outputs [7–9]. Hypothesized upstream controllers of the observed experimental data are drawn from those computations to be included into the network.

In the final step of network construction, the content and connectivity is subject to a terminal round of manual review. Ultimately, this three-step methodology results in computationally optimized network models whose edges are supported by published literature [10].

Data

CDK Inhibitor-Treated Normal Human Bronchial Epithelial Cells

NHBE cells were treated with the CDK4/6 inhibitor PD-0332991 (IC50 = 0.011 μmol/L for CDK4 and 0.016 μmol/L for CDK6). Specifically, cells were treated in vitro with media alone or media with 1 μM CDK inhibitor, for 24 hours, after which CDK inhibitor-treated cells were washed in media with or without the same concentration of CDK inhibitor, while control cells were washed in media alone. Cells were collected 2, 4, 6 and 8 hours after washing (3 petri-dishes per time point), and total RNA was extracted and hybridized to Affymetrix U133 Plus 2.0 microarrays. Data is available in Array Express, accession number E-MTAB-1272.

Rat 28-Day cigarette smoke inhalation

Outbred Sprague-Dawley rats were obtained from Charles River, France. The age of the rats at the start of the inhalation period was 7 weeks. The body weights were within 20% of the mean weight for each sex. The rats were nose-only exposed to main stream smoke (MS) or to filtered, conditioned air (sham exposure group) for 6 hours /day, 7 days/week for 28-days. Each group was exposed in a separate exposure chamber. The 28-day exposure period is defined in draft OECD guideline 412 (2005). The target MS concentrations were 8, 15, or 23 μg nicotine/l for the groups exposed to the reference cigarette 3R4F. A 5-day dose-adaptation regimen was applied at the start of the inhalation period. On study days 1 and 2, the rats were exposed to 1/3 of the target concentrations. On study days 3 and 4, the rats were exposed to 2/3 of the target smoke concentrations. As from day 5 on, the rats were exposed to the target concentrations. 3R4F cigarettes were smoked according to Health Canada Intensive Smoking Protocol (Health Canada, 1999) whereby MS was diluted with filtered, conditioned air to the target nicotine concentrations. Steady-state blood carboxyhemoglobin concentration, respiratory physiology parameters and representative nicotine metabolites in the urine were determined (data not shown). Food consumption and body weight, and hematological, clinical-chemical, gross pathological, histopathological, and pulmonary inflammation parameters were determined to characterize the biological activity of the smoke. The study was conducted in compliance with the OECD Principles on Good Laboratory Practices (as revised in 1997).

TNF exposure of RLAK cell

NRBE cells (normal rat bronchial epithelial cells) were either treated with a vehicle control (sham) or TNFα at concentrations of 0.1 (very low), 1 (low), 10 (medium), or 100 (high) ng/ml. Normal rat bronchial epithelial cells (Lonza Walkersville, Inc.)

were cultured in standard growth medium (Clonetics medium, Lonza Walkersville, Inc.). Cells were harvested after the desired treatment length (30 minutes, 2 hours, or 24 hours). Cells were immediately put on ice. Each experimental group contains 5 biological replicates. Data are available in Array Express, accession number E-MTAB-1311.

Gene expression profiling and analysis

Tissue Preparation

Dissection took take place directly after the last exposure. Left and right lungs were separately snap-frozen immediately after the preparation. To collect specific cellular structures of the lung (i.e., parenchyma and airways), Laser Capture Microdissection (LCM) was performed (PALM Microbeam, Carl Zeiss Microscopy GmbH, Jena). To this end, 20 μm slices were produced and mounted on special membrane slides (Zeiss, "MembraneSlide", 1.0 PEN'). For collecting the parenchyma material, only slices which were enriched for parenchyma (mainly at the exterior side of the lung) were used. As airway-conducting material, the main-bronchus, and the first main branching were taken. Only slices which were enriched for air-conducting structures (from the central part of the lung) were taken. The areas of interest (parenchyma, main bronchus) were exercised by laser micro-dissection and transferred into an RNAse-free reaction tube. Because the lasered areas were too large for the catapulting function of the microscope, the slices needed to be transferred with RNAse-free forceps (irradiated with UV light). Directly after the transfer, the tissue was lysed in 700 μl QIAzol lysis buffer (Qiagen). The samples were frozen on dry ice and stored at -80 degC until further processing.

RNA Preparation and Whole Genome Expression Arrays

RNA from the derived samples was isolated using miRNAeasy Mini Kit (Qiagen). The resulting RNA quality and quantity were analyzed using an Agilent 2100 Bio-Analyzer (Agilent, Waldbronn) and a NanoDrop ND-1000 (PeqLab, Erlangen). The starting material for the use of the GeneChip®3' IVT Express Kit (Affymetrix) was 100 ng RNA in 3 μl RNAase-free water. The procedure was carried out as described in the GeneChip®3' IVT Express Kit User Manual from Affymetrix. The Hybridization mix was incubated on GeneChip®Rat Genome 230 2.0 Arrays for 16 hours. Further processing was performed as described in the GeneChip®Expression Analysis Technical Manual with Specific Protocols for using the GeneChip Hybridization, Wash and Stain Kit Affymetrix. After hybridization, the arrays were inserted in the GeneChip®Fluidics Station 450 of Affymetrix which was operated as described in the GeneChip®Fluidics Station User's Guide. The Fluidics Station was controlled by the GeneChip Command Console software. The probe array underwent an automated washing and staining protocol specific for GeneChip®Rat Genome 230 2.0 Arrays. The arrays were further analyzed in the Affymetrix®Expression Console software application using the MAS5 algorithm to create CHP files and checked for several quality parameters provided in the Affymetrix data analysis software package. Command Console Software (Affymetrix) was used to automatically grid the DAT files and create the CEL files (probe cell intensity data). Data processing was implemented in the R statistical environment. Raw RNA expression data were

analyzed using the affy and gcrma packages of the Bioconductor suite of microarray analysis tools available in the R statistical environment. Robust Microarray Analysis (RMA) background correction and quantile normalization were used to generate probe set expression values.

References

1. Selventa: The BEL Language Portal. http://www.openbel.org
2. Falcon, S., Gentleman, R.: Hypergeometric testing used for gene set enrichment analysis. Bioconductor Case Studies, 207–220 (2008)
3. Subramanian, A., Tamayo, P., Mootha, V.K., Mukherjee, S., Ebert, B.L., Gillette, M.A., Paulovich, A., Pomeroy, S.L., Golub, T.R., Lander, E.S., Mesirov, J.P.: Gene set enrichment analysis: a knowledge-based approach for interpreting genome-wide expression profiles. Proc. Natl. Acad. Sci. U.S.A. **102**(43), 15545–15550 (2005)
4. Selventa: Reverse Causal Reasoning Methods Whitepaper. http://www.selventa.com/technology/white-papers
5. Liao, J.C., Boscolo, R., Yang, Y.-L., Tran, L.M., Sabatti, C., Roychowdhury, V.P.: Network component analysis: reconstruction of regulatory signals in biological systems. Proceedings of the National Academy of Sciences **100**(26), 15522–15527 (2003)
6. Catlett, N.L., Bargnesi, A.J., Ungerer, S., Seagaran, T., Ladd, W., Elliston, K.O., Pratt, D.: Reverse causal reasoning: applying qualitative causal knowledge to the interpretation of high-throughput data. BMC Bioinformatics **14**(1), 340 (2013)
7. Kumar, R., Blakemore, S.J., Ellis, C.E., Petricoin, E.F., Pratt, D., Macoritto, M., Matthews, A.L., Loureiro, J.J., Elliston, K.: Causal reasoning identifies mechanisms of sensitivity for a novel AKT kinase inhibitor, GSK690693. BMC Genomics **11**, 419 (2010)
8. Laifenfeld, D., Gilchrist, A., Drubin, D., Jorge, M., Eddy, S.F., Frushour, B.P., Ladd, B., Obert, L.A., Gosink, M.M., Cook, J.C., Criswell, K., Somps, C.J., Koza-Taylor, P., Elliston, K.O., Lawton, M.P.: The role of hypoxia in 2-butoxyethanol-induced hemangiosarcoma. Toxicol. Sci. **113**, 254–266 (2010)
9. Smith, J.J., Kenney, R.D., Gagne, D.J., Frushour, B.P., Ladd, W., Galonek, H.L., Israelian, K., Song, J., Razvadauskaite, G., Lynch, A.V., Carney, D.P., Johnson, R.J., Lavu, S., Iffland, A., Elliott, P.J., Lambert, P.D., Elliston, K.O., Jirousek, M.R., Milne, J.C., Boss, O.: Small molecule activators of SIRT1 replicate signaling pathways triggered by calorie restriction in vivo. BMC Syst Biol **3**, 31 (2009)
10. Schlage, W.K., Westra, J.W., Gebel, S., Catlett, N.L., Mathis, C., Frushour, B.P., Hengstermann, A., Van Hooser, A., Poussin, C., Wong, B., Lietz, M., Park, J., Drubin, D., Veljkovic, E., Peitsch, M.C., Hoeng, J., Deehan, R.: A computable cellular stress network model for non-diseased pulmonary and cardiovascular tissue. BMC Syst Biol **5**, 168 (2011)

Supplementary Figures

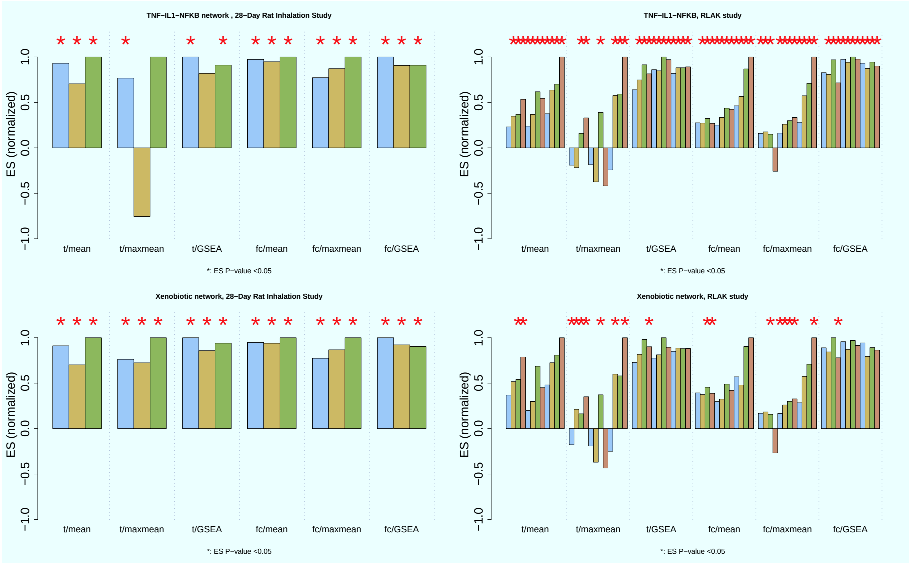

Figure S1 Forward FCS approach results using the 28 Day Inhalation study and the TNF-induced response in RLAK cells study, using both TNF-IL1α-TLR-NFκB and the xenobiotic metabolism response networks. Several combinations of gene level statistics and geneset enrichment statistics are compared. Among those, the combination "fc / maxmean" is the method leading to the best qualitative and quantitative results, but still predicting some activation of the xenobiotic response metabolism for the TNFα-induced treatment in RLAK cells (bottom right panel). Our method is the only one exhibiting the expected qualitative and quantitative pattern. A "*" indicates a significant enrichment (P-value¡0.05). ES is teh enrichemnt score of each methods normalized to its maximum value. See main text, method section for further details.

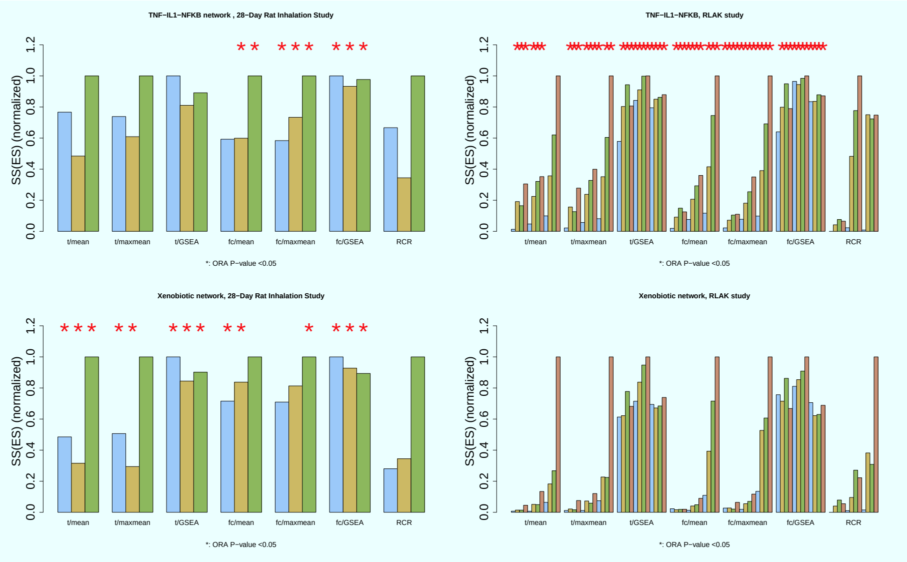

Figure S2 Backward FCS/ORA approach: each node in the functional layer having connections to the transcript layer is scored either by classical FCS approaches based several combination of gene level and geneset enrichment statistics, or RCR. A binomial ORA test is then performed to assess the enrichment of the two-layer network. A quantitative score is derived as the sum of square (SS) of the enrichment statistics of each nodes scored. For RCR $-log_{10}(p - value)$ is used. While again the combination "fc / maxmean" leads to the best results, it fails at identifying the perturbation of the Xenobiotic network at low and medium doses. A "*" indicates a significant enrichment (P-value< 0.05). $SS(ES)$ denotes the sum of square enrichment scores (normalized to its maximum value for each method). Further details are described in the main text, method section.

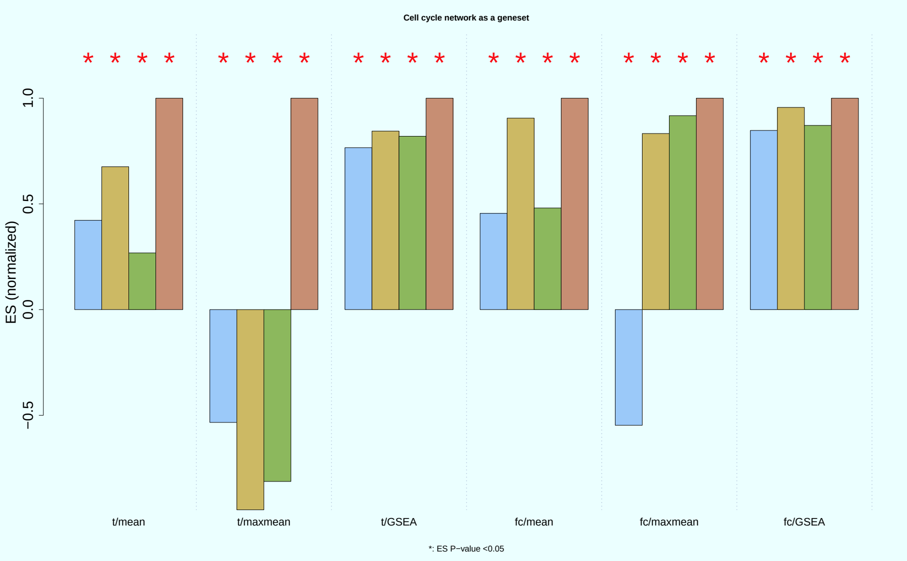

Figure S3 Forward FCS approach results using the CDK-inhibitor study, using the cell cycle networks. Several combination of gene level statistics and geneset enrichment statistics are compared. Among those, the combination fc / maxmean is the method leading to the best qualitative and quantitative results. A "*" indicates a significant enrichment (P-value< 0.05). ES is teh enrichemnt score of each methods normalized to its maximum value. See main text, method section for further details.

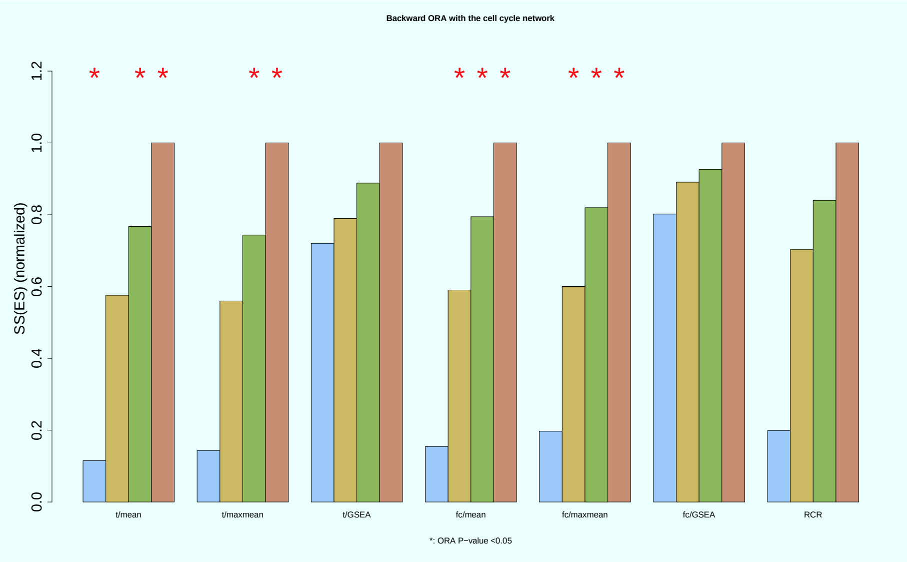

Figure S4 Backward FCS/ORA approach: each node in the functional layer having connections to the transcript layer is scored either by classical FCS approaches based several combinations of gene level and geneset enrichment statistics, or RCR. A binomial ORA test is then performed to assess the enrichment of the two-layer network. A quantitative score is derived as the sum of square (SS) of the enrichment statistics of each nodes scored. For RCR $-log_{10}(p - value)$ is used. While again the combination "fc / maxmean" leads to the best results, while the score for 2h INH+GM is negative. A "*" indicates a significant enrichment (P-value< 0.05). $SS(ES)$ denotes the sum of square enrichment scores (normalized to its maximum value for each method). Details can be found in the main text, method section.

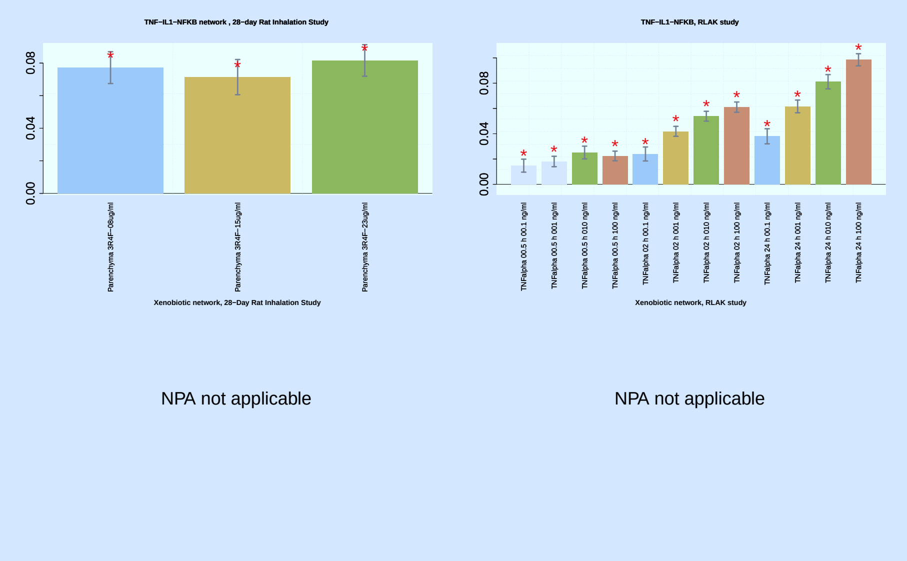

Figure S5 NPA (Martin et al. 2012) results using the 28 Day Inhalation study and the TNF-induced response in RLAK cells study, using both TNF-IL1α-TLR-NFκB. The method is not applicable to the xenobiotic network as this model is not causally consistent. The qualitative pattern of the NPA method, when applicable, is matching with the expectations. While behaving quantitatively for the RLAK study, it fails to quantify the dose response in the 28-day rat inhalation study. Again, TopoNPA method is one exhibiting both the expected qualitative and quantitative pattern. A "*" indicates a significant enrichment (P-value< 0.05).

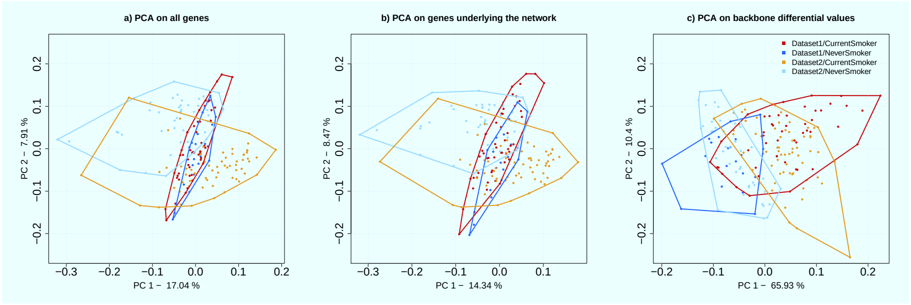

Figure S6 Principal Component Analysis (PCA) of the backbone values of the xenobiotic metabolism response network . Panel c) shows the alignment of the phenotype with the first component as opposed to the PCA using all gene expression data (panel a)), or only gene expression data from the genes underlying the network (panel b)).

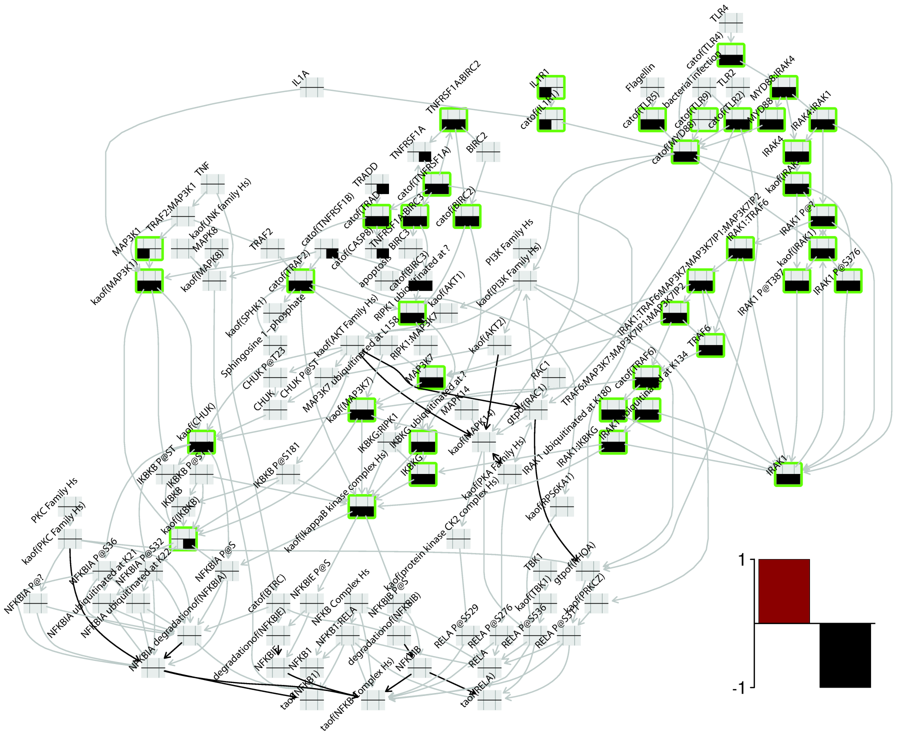

Figure S7 Network leading nodes of TNF-IL1α-TLR-NFκB network for the comparison of IFX responders vs. IFX non-responders, before (left bar) and after (right bar) treatment. Values are -1 (respectively +1) if the node is a leading node and its differential backbone value is negative (respectively positive), or 0 if it is not a leading node. Nodes circled in green are the leading nodes of the perturbation of the network from the comparison shown in Figure 6 of the main text.

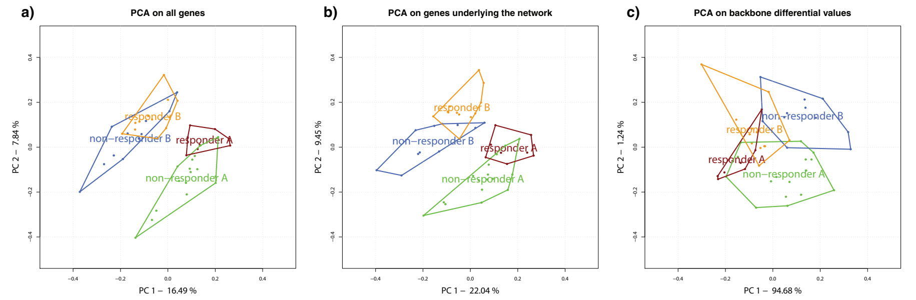

Figure S8 Principal Component Analysis (PCA) of the backbone values of the TNF-IL1α-TLR-NFκB network . Panel c) shows the alignment of the phenotype with the first component as opposed to the PCA using all gene expression data (panel a)), or only gene expression data from the genes underlying the network (panel b)). The first cohort (A) of IFX responders (respectively IFX non-responders) are displayed in brown (respectively green) and the second cohort (B) of IFX responders (respectively IFX non-responders) are displayed in orange (respectively blue).

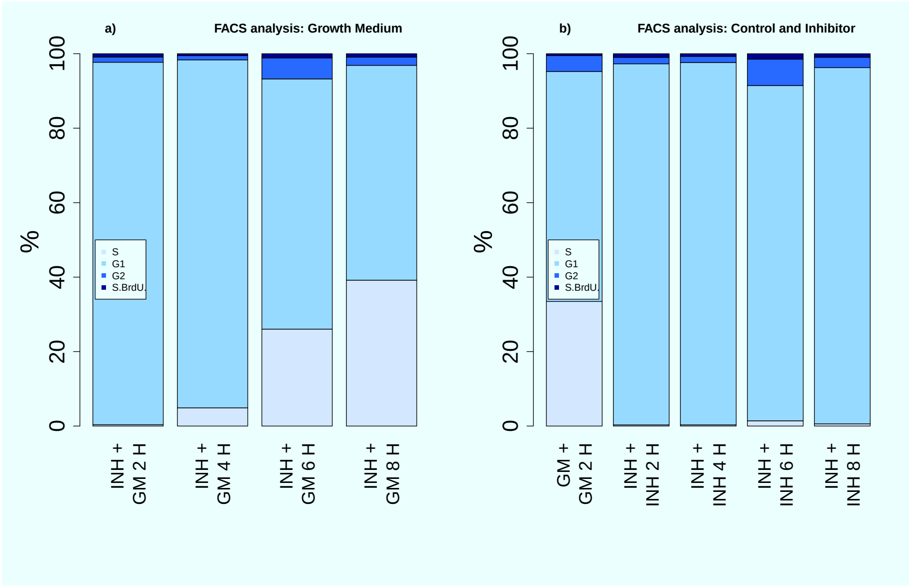

Figure S9 Results of the Fluorescence Activated Cell Sorting (FACS) analysis showing the re-entry in S-phase after removal of the inhibitor (panel a)). Control and inhibited cell sorting results are shown in panel b). The percentage of cell re-entering in S-phase is increasing with time.

Supplementary Tables

Table S1 Prediction sensitivities and specificities for the two datasets, shown with standard errors for cross-validation results, GSE7895 (D_1), GSE19667 (D_2). The predictions of the samples from the dataset D_j based on the model trained on the dataset D_i are reported in the columns $D_i \rightarrow D_j$. While not systematically having the best cross-validaiton performance, the predictors based on the backbone values show are more robust behavior when predicting one dataset based on the model trained on the other one. The mean of the G-performances ($= \sqrt{Sp \cdot Se}$) over the two independant test sets are shown in the rightmost column and is highlighted if > 0.7. The best UBE based models is chosen based on the mean cross-validation G-performance for the two datasets. The algorithms based on the backbone values leads systematically to good performances.

Type	Method	CV (D_1/D_2)		$D_1 \rightarrow D_2$		$D_2 \rightarrow D_1$		Mean G-perf
		Se	Sp	Se	Sp	Se	Sp	Test sets
All Genes	tForwardLd	0.95 ± 0.06 / 0.86 ± 0.05	0.93 ± 0.04 / 0.92 ± 0.03	1.00	0.87	0.69	1.00	**0.88**
	NSC	0.92 ± 0.07 / 0.94 ± 0.04	0.96 ± 0.03 / 0.93 ± 0.03	1.00	0.00	0.98	0.00	0.00
	RF	0.75 ± 0.11 / 0.96 ± 0.04	1.00 ± 0.01 / 0.97 ± 0.02	1.00	0.31	0.98	0.95	**0.76**
	SVM	0.84 ± 0.10 / 0.94 ± 0.05	0.98 ± 0.02 / 0.96 ± 0.02	1.00	0.00	1.00	0.00	0.00
Gene in transcript layer	tForwardLd	0.95 ± 0.06 / 0.89 ± 0.04	0.92 ± 0.04 / 0.93 ± 0.03	0.97	0.91	0.61	0.85	**0.83**
	LDA	0.92 ± 0.05 / 0.94 ± 0.03	0.80 ± 0.10 / 0.97 ± 0.03	0.62	0.27	0.86	0.40	0.50
	NSC	0.96 ± 0.03 / 0.92 ± 0.03	0.93 ± 0.05 / 0.95 ± 0.04	1.00	0.00	1.00	0.00	0.00
	RF	0.87 ± 0.09 / 0.96 ± 0.04	1.00 ± 0.00 / 0.97 ± 0.02	0.98	0.58	0.94	1.00	**0.86**
	SVM	0.88 ± 0.07 / 0.95 ± 0.04	0.98 ± 0.02 / 0.95 ± 0.02	1.00	0.00	0.78	0.15	0.17
UBE down-stream genes	CORG + LDA	0.97 ± 0.03 / 0.94 ± 0.03	0.90 ± 0.07 / 0.94 ± 0.04	0.83	0.36	0.61	0.55	0.56
	CORG + NSC	0.98 ± 0.02 / 0.95 ± 0.03	0.93 ± 0.05 / 0.96 ± 0.04	0.97	0.00	0.67	0.30	0.22
	Best LDA (8-Methyl-IQX)	0.96 ± 0.03 / 0.95 ± 0.03	0.88 ± 0.07 / 0.96 ± 0.03	0.9	0.80	0.80	1.00	**0.89**
	Best NSC (8-Methyl-IQX)	0.96 ± 0.02 / 0.86 ± 0.03	0.92 ± 0.03 / 0.98 ± 0.02	0.98	0.76	0.86	1.00	**0.90**
Backbone values	tForwardLd	0.97 ± 0.04 / 0.90 ± 0.04	0.95 ± 0.06 / 0.97 ± 0.02	0.8	0.82	0.84	0.90	**0.86**
	LDA	0.96 ± 0.03 / 0.92 ± 0.03	0.94 ± 0.06 / 0.98 ± 0.02	0.89	0.80	0.84	0.90	**0.86**
	NSC	0.93 ± 0.03 / 0.93 ± 0.03	0.81 ± 0.10 / 0.92 ± 0.04	0.95	0.87	0.94	0.90	**0.91**
	RF	0.93 ± 0.03 / 0.91 ± 0.03	0.80 ± 0.10 / 0.91 ± 0.05	0.97	0.73	0.88	0.85	**0.85**
	SVM	0.93 ± 0.04 / 0.93 ± 0.03	0.88 ± 0.07 / 0.91 ± 0.05	0.98	0.62	0.88	0.90	**0.83**

Table S2 Prediction sensitivities and specificities, shown with standard errors for cross-validation results, for the two cohorts,A and B. The predictions of the samples from the cohort A (B respectively) based on the model trained on the cohorts B (A respectively) are reported in the columns $A \rightarrow B$ ($B \rightarrow A$ respectively). The mean of the G-performances ($= \sqrt{Sp \cdot Se}$) over the two independant test sets are shown in the rightmost column and is highlighted if > 0.7. The best UBE based models is chosen based on the mean cross-validation G-performance for the two datasets. The algorithms based on the backbone values leads to good performances for a majority of algorithms.

Type	Method	CV (A/B)		$A \rightarrow B$		$B \rightarrow A$		Mean G-perf
		Se	Sp	Se	Sp	Se	Sp	Test sets
All Genes	tForwardLd	0.38 ± 0.15 / 0.63 ± 0.14	0.80 ± 0.11 / 0.65 ± 0.15	0.50	0.82	1.00	0.88	**0.79**
	From Arijs, 2009	accuracy: 0.92/0.91		0.25	1	accuracy: 0.71		na
	RF	0.20 ± 0.15 / 0.82 ± 0.13	0.88 ± 0.09 / 0.73 ± 0.14	0.25	0.91	0.62	0.75	0.58
	SVM	0.52 ± 0.16 / 0.78 ± 0.13	0.85 ± 0.10 / 0.69 ± 0.14	0.42	0.82	0.62	0.75	0.63
	NSC	0.48 ± 0.16 / 0.78 ± 0.12	0.80 ± 0.11 / 0.58 ± 0.16	0.67	1.00	0.69	0.88	**0.80**
Gene in transcript layer	tForwardLd	0.50 ± 0.15 / 0.68 ± 0.14	0.83 ± 0.10 / 0.62 ± 0.15	0.67	1.00	0.88	0.69	**0.80**
	LDA	0.43 ± 0.16 / 0.90 ± 0.09	0.84 ± 0.10 / 0.65 ± 0.15	0.42	0.82	0.88	0.62	0.66
	NSC	0.60 ± 0.16 / 0.88 ± 0.10	0.79 ± 0.11 / 0.58 ± 0.16	0.75	0.73	0.88	0.56	**0.72**
	RF	0.28 ± 0.16 / 0.85 ± 0.11	0.88 ± 0.09 / 0.71 ± 0.14	0.33	0.82	0.88	0.69	0.65
	SVM	0.52 ± 0.17 / 0.77 ± 0.13	0.86 ± 0.11/ 0.69 ± 0.13	0.42	0.82	0.88	0.69	0.68
UBE down-stream genes	CORG + LDA	0.33 ± 0.15 / 0.73 ± 0.13	0.68 ± 0.12 / 0.62 ± 0.16	0.25	0.64	0.75	0.69	0.56
	CORG + NSC	0.62 ± 0.15 / 0.90 ± 0.09	0.78 ± 0.11 / 0.60 ± 0.16	0.50	0.82	0.88	0.69	**0.71**
	Best LDA (MAP3K1)	0.45 ± 0.14 / 0.98 ± 0.05	0.78 ± 0.11 / 0.69 ± 0.15	0.58	0.91	1.00	0.75	**0.80**
	Best NSC (catof(TLR2))	0.85 ± 0.12 / 0.92 ± 0.02	0.80 ± 0.11 / 0.56 ± 0.15	0.75	1.00	1.00	0.69	**0.85**
Backbone values	tForwardLd	0.53 ± 0.14 / 0.82 ± 0.13	0.75 ± 0.12 / 0.75 ± 0.13	0.50	0.64	1.00	0.62	0.68
	LDA	0.75 ± 0.11 / 0.75 ± 0.13	0.76 ± 0.14 / 0.75 ± 0.11	0.50	0.73	0.88	0.56	0.65
	NSC	0.98 ± 0.05 / 0.98 ± 0.02	0.75 ± 0.12 / 0.67 ± 0.15	0.92	0.82	1.00	0.62	**0.83**
	SVM	0.78 ± 0.11 / 0.78 ± 0.13	0.40 ± 0.17 / 0.77 ± 0.13	0.91	0.50	0.69	0.88	**0.73**
	RF	0.75 ± 0.12 / 0.82 ± 0.11	0.62 ± 0.15 / 0.78 ± 0.13	0.82	0.75	0.75	0.88	**0.80**
